# Individual variation in the habitat selection of upstream migrating fish near a barrier

**DOI:** 10.1186/s40462-023-00414-0

**Published:** 2023-08-07

**Authors:** Rachel Mawer, Stijn P. Bruneel, Ine S. Pauwels, Jelger Elings, Eliezer Pickholtz, Renanel Pickholtz, Matthias Schneider, Johan Coeck, Peter L. M. Goethals

**Affiliations:** 1 Department of Animal Sciences and Aquatic Ecology, Ghent University, Ghent, Belgium; 2 SJE Ecohydraulic Engineering, Backnang, Germany; 3 Research Institute for Nature and Forest (INBO), Brussels, Belgium; 4 Independent Researcher, East Brunswick, NJ USA; 5 School of Zoology, George S. Wise Faculty of Life Sciences, Tel Aviv University, Tel Aviv, Israel; 6 The Interuniversity Institute for Marine Sciences of Eilat, Eilat, Israel

**Keywords:** Fish migration, Step selection functions, Fine-scale telemetry, 2D acoustic telemetry, Fish passage

## Abstract

**Background:**

Migration is a vital element of the life cycle of many freshwater fish species but is increasingly hampered globally by riverine barriers. Fish passes are a common approach to enable migration past barriers but are often ineffective. More knowledge is required on fish behaviour as they approach barriers such as habitat preferences.

**Methods:**

We evaluate the habitat selection of two upstream migrating fish species, barbel *Barbus barbus* and grayling *Thymallus thymallus*, at a hydropower plant in southern Germany, considering individual variation and population trends. Fish were tracked via fine-scale 2D acoustic telemetry in 2018 during their spawning migration. Step selection functions were used to evaluate selection of hydraulic parameters by the fish for a time step of 20 s. Exploratory models were built via model selection for each individual fish, to evaluate the extent of individual variation in model structure. A population model was developed for each species by averaging coefficients from individual models to describe general trends. The extent of individual variation was determined and confidence intervals for the population model coefficients were calculated.

**Results:**

Fish varied greatly in individual model structure though common terms were apparent in both species, such as depth, flow velocity, the angular difference between fish and velocity, and the logarithm of the step length. Final population models for barbel included several parameters describing habitat selection and displacement. Barbel selected for faster flows, deeper water, and higher spatial velocity gradients. In addition, they selected to move more with the flow than against. Interactions were also present between habitat parameters, suggesting selection is context dependent. Barbel movement speed also changed with depth, flow velocity and spatial velocity gradient. With grayling, terms often had contrasting effects among individuals and thus general trends could not be distinguished for most terms.

**Conclusion:**

Our findings demonstrate habitat selection by upstream migrating fish approaching a fish pass and differences in individual selection which may have an impact on barrier management. Step selection functions are a promising approach and can provide useful insight into habitat selection and movement by migrating freshwater fish in an altered river system.

**Supplementary Information:**

The online version contains supplementary material available at 10.1186/s40462-023-00414-0.

## Introduction

Migration is a widespread phenomenon in freshwater fish taxa [1, 2]. Fish migrations occur across a wide spatiotemporal scale, from small-scale migrations within the same river to the colossal migrations undertaken by thousands of eels every year between rivers and the sea. Migrations serve many purposes, such as to exploit new habitat for spawning or growth, ultimately increasing overall reproductive output [1]. Migratory disruption can be catastrophic for animal populations [3]. For migration, fish are dependent on natural river flows for migration cues [2] and unfragmented rivers for connectivity between habitats [4]. However, free flowing rivers are growing increasingly rarer with the construction of anthropogenic barriers [5].

Over the last century, riverine barriers have grown in number, blocking rivers and hampering freshwater fish migration [4, 6]. Along a river, barriers can be frequent. For example in Europe, barriers occur at a range of 0.74 barriers per km on average [5], with subsequent barriers along a river resulting in cumulative impacts [7]. Barriers impacts upon freshwater fish are broad, from direct obstructions to altering the natural flow hydraulics of a river [8]. Firstly, barriers physically impede movement and migration. A barrier can completely prevent fish completing their migration, cause injury or mortality, or introduce a migration delay, which increases energy expenditure in the migration and results in lower reproductive output for spawning. For example, salmon smolt had lower survival in a regulated river compared to unregulated [9]. The negative impacts of barriers has been known for centuries [10] and in recent decades increased effort has been directed towards fish passage. The resulting impact of barriers on fish populations has been drastic. Distributions of migratory species have reduced along with local extinctions [11] and barriers have contributed to a 76% overall decline in freshwater fish populations since the 1970s [12]. The true extent of barriers, both large and small, in river systems is often unrecorded and their resulting impacts may be underestimated [5, 13]. Despite the impact more barriers are planned, further reducing river connectivity [5, 6].

Steps have been taken to mitigate the impact of barriers upon freshwater fish. Fish passes are a widely implemented choice for mitigating barrier impacts, providing an alternative route to migrating fish in an attempt to reconnect fragmented river systems [4]. A range of different fish passes exist with species-specific advantages and disadvantages. While a promising concept and a success in some cases, fish passes have variable success and a historic focus on salmonids has often resulted in unsatisfying success rates for other species. For example, in meta analyses of fish passage, upstream and downstream passage had a mean efficiency of 41.7% and 68.5% respectively, with salmonids having significantly higher passage efficiency than non-salmonids [14] and efficiency can vary greatly with pass design [15]. With the current state of river fragmentation and future barrier proposals [5], there is growing need to improve fish passage facilities for the wider fish community.

One area through which passage failure can arise is by fish pass attractiveness [16]. In order to pass, a fish first must be able to locate the fish pass yet in the highly modified environment around a barrier that may be challenging. Hydraulic cues guide fish during their migration [2, 4] yet such cues may be weak near barriers. Moreover, such responses to cue may also vary individually: individual variation is common in animal spatial usage and fish passage. For example, Capra et al. [17] found strong individual variation in habitat selection in three fish species in an altered riverine environment. Ignoring individual variation could lead to false conclusions [18, 19]. Moreover, migrating fish follow the river flow and the flow through the fish pass is often insufficient compared to the discharge from the barrier: the flow from the pass is drowned out by discharge through the barrier and fish cannot find the pass [16]. Much work has been conducted on improving pass attractiveness, for example via supplementary flows [16, 20]. There is further need to study fish behaviour and movement through the altered environment as they approach the barrier and find the pass [16].

Technological advances in recent decades have led to a proliferation of data on fish movement in the vicinity of barriers, alongside hydraulic data. Acoustic telemetry can provide information on fish positions as fine as every second [21] while fine-scale hydrodynamic models can supply hydraulic data on sub-meter scales [22, 23]. Together, hydraulic and positional data provide an insight into the conditions experienced by a fish as it moves through its environment. Such data can be analysed to determine whether fish exhibit any preferences in habitat selection – do fish prefer deeper waters? Faster flows? – which can then guide management or fish pass design.

Step selection functions (SSFs) are one of the techniques to model habitat preferences of moving animals [24] and are implementable via the ‘amt’ package in R, with instructions and example code available [25]. SSFs statistically compare habitat at steps taken by the animal to potential alternative steps the animal could have taken [25]. Through doing so, SSFs provide estimates for habitat preference and identify which habitat parameters may be selected by animals. SSFs have been widely applied to terrestrial ecosystems [24] but their use in aquatic environments is still rare (and restricted to the marine environment [26, 27]). Moreover, current applications are for data on comparatively coarser scales than fish passage approach: for example, SSFs have been applied with fixed time steps of > 15 min to hours [24] whereas a sub-minute scale is of interest for navigating fish. To our knowledge, there are currently no peer-reviewed publications applying SSFs to freshwater fish in rivers.

In the context of fish passage science and alongside technological advances, SSFs can provide insight into fish habitat preference as they navigate towards a barrier and fish pass. In addition, SSFs could improve understanding of the extent of individual variability when approaching a fish pass, supporting barrier mitigation [16]. If we understand which habitats fish prefer and select for and know how the hydraulic environment changes under different flow discharges or hydropower plant regimes, we can visualise how fish usage may change under different conditions. This in turn could help determine whether fish may move towards a fish pass or not. Moreover, outputs from SSFs could be fed into mechanistic models for predicting fish passage success, such as individual based models [28, 29], with further benefits for prediction across different sites and set ups. SSFs hold great potential for analysing the wealth of environmental and positional data available on migrating fish.

Here, we present the application of SSFs to upstream migrating barbel (*Barbus barbus)* and grayling (*Thymallus thymallus)* at a hydropower plant in southern Germany. The aims of this study were to identify habitat preferences of migrating fish as they approach the fish pass and to quantify individual and species-specific differences. We examined the difference in model structure between species and individuals. Coefficients from individual models were averaged to form population models, describing trends for each species. Our specific goal was to provide first insight and preliminary results on habitat selection by the two species in this context, to direct future work.

## Methods

### Study site and acoustic array

**Fig. 1:**
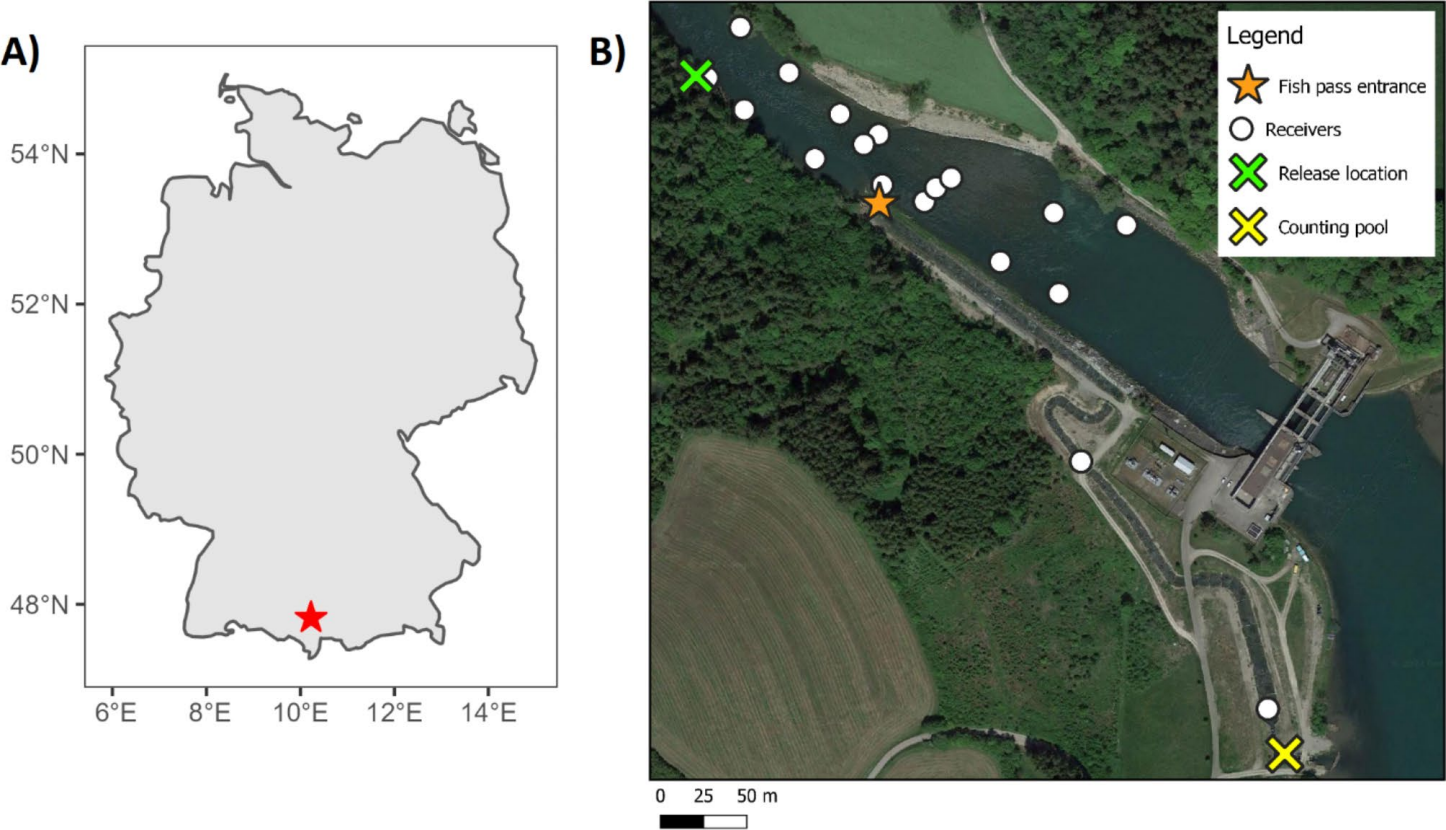
The study site, Altusried hydropower plant. (**A**) A map of Germany with the study site marked by a red star. (**B**) A map of the receiver array with receivers represented by white dots. Two receivers were in the fish pass and a third receiver (not pictured) was positioned downstream of the main array. The fish pass entrance is marked with an orange star. The green cross marks the release location of tagged fish and the yellow cross shows the location of the counting pool, where many fish were initially caught. Water flows from the bottom right of the map to the top left

The Altusried hydropower plant (HPP) is located on the River Iller in southern Germany (Fig. 1A). The HPP has a capacity of 7.8 MW and two Kaplan turbines, and has been in operation since 1961. In 2014, a fish pass was built. The lower part of the fish pass is a nature-like slot pass while the upper part is a vertical slot pass. The fish pass is 525 m in length with a design flow of 1 ms^− 1^. The gradient of the fish pass is 0.8–2.5% and maximum depth in the fish pass is 0.8 m. The area downstream of the HPP ranges in depth from 0.001 to 7.56 m (mean 2.50 m across discharges) with flow velocity ranging from 0 m.s^− 1^ to 2.24 m.s^− 1^ (mean 0.35 m.s^− 1^). During the analysed periods, river flow discharge had a mean ± standard deviation of 57.01 ± 24.11 m^3^.s^− 1^. A 2D acoustic array was installed downstream of the hydropower plant (Fig. 1B) using 16 180kHZ HR2 VEMCO receivers. Two receivers were also positioned in the fish pass (halfway and upstream) as well as a single receiver downstream of the study site.

### Data collection

#### Fish tagging

In total, 22 barbel (*Barbus barbus*; TL: 498 ± 73 mm; weight: 1356 ± 592 g) and 25 grayling (*Thymallus thymallus*; TL: 367 ± 56 mm; 630 ± 270 g) were caught and tagged between March 28th and May 29th 2018 (Supplements: table S1). Barbel were caught between May 17th and May 29th while grayling were caught between March 28th and 11th April. Fish were caught via electrofishing in the study site (grayling n = 14), electrofishing in the fish pass (barbel n = 2), or in the counting pool of the fish ladder (barbel n = 20, grayling n = 10). Capture information was not recorded for one grayling. The sex of a minority of fish was identified and recorded (Supplements: table S1). Fish were anaesthetised with phenoxy-ethanol (10 ml per 50 l water) and a VEMCO V9 acoustic tag (rbi PPM: 50–70 s; rbi HR: 1.1–1.3 s; weight 2 g in water) was surgically implanted into the abdomen according to Thorstad et al. [30]. After tagging, fish recovered in another aerated tank and were released downstream of the study site (next to the most downstream receiver, Fig. 1B) as soon as they were able to swim (2–11 min after tagging). Fish were tracked from tagging until the receiver array was removed on August 22nd 2018.

#### Environment data and hydraulic modelling

Hydraulic data were collected and modelled with the 2D hydrodynamic numerical model Hydro_AS-2D [31] for flow discharge scenarios of 10 to 80 m^3^s^− 1^ (in 10 m^3^s^− 1^ increments). The hydraulic model provided data on flow velocity, depth, spatial velocity gradient (SVG) and the direction of flow and SVG at a resolution of 0.5 × 0.5 m for an area up to around 500 m downstream of the HPP. Resulting environmental data is shown in Table 1 and rasters of the five environmental variables are shown in the supplementary file “Supplements”.

**Table 1:** Parameters included in the step selection functions. Spatially varying parameters are environmental parameters that are not uniform across the study site at a point in time. Movement parameters are parameters describing elements of the fish’s movement or position. Other environmental parameters are external parameters that are uniform across the study site (e.g. temperature does not change between true and null steps in the step selection function): these parameters were only included via interactions

Parameter name	Abbreviation	Definition
*Spatially varying parameters, available at the start and end of steps*		
Flow velocity	WV	Flow velocity, in meters per second
Spatial velocity gradient (SVG)	SVG	The spatial change of flow velocity between cells in the hydraulic model, in meters per second per meter
Water depth	D	Depth of water, in meters
Difference between flow angle and fish’s angle	DiffVang	The angular difference between the direction of the flow velocity and the direction of the fish (where 0° is north). Modelled as the cosine
Difference between SVG angle and fish’s angle	DiffSVGang	The angular difference between the direction of the SVG and the direction of the fish (where 0° is north). Modelled as the cosine
*Movement parameters*		
Step length	SL	Distance travelled in a step, modelled as its logarithm
Turning angle	TA	Angle at which a fish has turned from the preceding step, modelled as the cosine
*Other parameters*		
Temperature	Temp	Recorded by each receiver every 15 min. Median value taken for the whole study site. Temperature record closest in time to a fish position was used.
Time of day	TOD	Points categorised as dawn, day, dusk or night, based on nautical twilight.

### Data processing

Resulting telemetry data were filtered to retain tracks when fish approached the pass during the spawning migration. Specifically, fish locations were analysed to identify when fish came within 10 m of a fish pass. If such a detection occurred within 2 h of a previous detection near the fish pass it was discarded, to remove cases where fish remained in the area for extended periods. From remaining detections, the preceding 60 min (if available) were selected as the approach. Tracks were checked and rejected if: over 90% of detections were within 15 m of the pass entrance; if the track was less than 30 min in length; or if there were less than 10 detections over the period. Resulting tracks were constrained to within the known migration period for the species, based on previous analysis of counting pool data in the River Iller [32]. Data for both species were constrained between date of tagging and June 16th for barbel and April 16th for grayling. Some fish approached the fish pass on multiple occasions and repeated approaches were included in the analysis.

Step selection functions (SSFs) require regular time steps in their data [25] which is a constraint of acoustic studies where regularised data are rare. Raw positions for the fish were as fine as around 1.1 s between detections but varied greatly. Thus, tracks for each approach were interpolated for 20 s intervals using the ‘crawl’ package in R [33]. A 20 s time step was chosen due to a trade-off between study goals and data. Our aim was to study fine-scale movements of fish but both positioning error and environmental data resolution dictated a relatively coarser resolution was needed: with the latter, at finer resolutions (e.g. 10 s) fish step lengths often did not exceed raster cell size. Where a gap in detections greater than 60 s existed (e.g. three times the time step), the track was split into segments with crawl applied to each segment separately: this approach was similar to Lamonica et al. [34] though they used a smaller gap. If a segment contained fewer than 10 detections, the segment was discarded. Resulting positions that were not within the extent of the study site were removed.

Initially, 100 random steps per true step were created from parametric distributions fitted to true step lengths and turning angles, using gamma and von Mises distributions respectively [24]. The function ‘random_steps()’ in the R package ‘amt’ was used to create random steps [25]. Random steps were filtered to remove those ending outside the river and environmental rasters. From the remaining pool of random steps, ten steps were selected at random for each true step. We further filtered tracks to retain tracks of interest, which were specifically those showing directed movement in the direction of the fish pass and did not begin near the pass (see supplementary file “Supplements”: “*Filtering tracks*”). We also removed tracks with fewer than two steps per minute on average. After processing, 87 tracks for 31 fish remained. Pass approach behaviour were extracted for 20 barbel and 11 grayling, ranging from one to nine fish pass approach tracks per fish (mean 2.8, median 2). Final tracks were compared to data from receivers within the pass to record which approaches resulted in successful entry into the pass (four tracks preceded detections in the ladder, two for barbel and two for grayling).

Environmental data were added to the start and end points of each step from 0.5 × 0.5 m resolution raster data produced via hydraulic models. Time of day (category: dawn, day, dusk or night) was also added to the data. The function ‘crepuscule’ from the R package ‘maptools’ [35] was used to identify dawn, dusk, sunrise and sunset times for each date during our study period and categorise time periods appropriately. We defined dawn as the period between the nautical dawn (sun 12° below the horizon) and sunrise, and dusk as the time between sunset and nautical dusk (sun 12° below the horizon). Dawn and dusk length ranged from 69.54 to 97.16 mins (mean dawn length 85.76 min, mean dusk length 85.98 min). Temperature data were collected by the receivers at 15 min intervals and the median value calculated. For each start and end points of a step, nearest median value temporally was assigned: temperature data did not vary spatially.

All data analyses were done in RStudio version 4.2.2 [36].

### Modelling

Step length was log transformed. All angles were modelled as their cosine to linearise the variables, which converted angles from being on a circular scale to linear scale between − 1 and 1. Values approaching 1 represent smaller angles. In addition, variables were standardised prior to modelling to place parameters onto a similar scale and enable comparison of effect sizes. Standardisation occurred by subtracting the mean value and dividing by standard deviation. To permit comparisons between fish, standardisation occurred using global means and standard errors for all fish and species.

We had two goals for modelling. Firstly, we wanted to be able to compare model structure between fish – was the movement of individual fish affected by different parameters? For this, we performed model selection on each fish individually. We adopted an explanatory modelling protocol [37] and started with a broad saturated model in order to determine terms explaining the data for each fish. Terms were checked for correlations: all Pearson’s correlation coefficients were below 0.5 in magnitude, except from between the same variable at the start and end of a step.

Conditional logistic regression models were fitted to the data for each fish, using the function ‘fit_issf()’ in the R package ‘amt’ [25]. Generalised linear modelling was used as preliminary non-linear models indicated linear relationships between parameters and habitat selection. Model selection followed backwards stepwise selection using Akaike’s Information Criterion (AIC) to select the most parsimonious model. Model terms were removed one-by-one. A more complex model was retained if it had an AIC of at least two units lower than the simpler model. Otherwise, the simpler model would be kept. Saturated model terms are shown in Table 2. We decided to test broadly whether terms did or did not explain movement of each fish. If a fish only had one time of day category in its data (e.g. all steps were in the same time period), terms including time of day interactions were not tested.

**Table 2:** Saturated model terms applied to every fish, split by the broader question. Where a fish only had tracks during one time of day period, all time of day terms were not included. Terms are abbreviated as per Table 1

Question	Terms
Habitat selection	WV (end) + D (end) + SVG (end) + DiffVang (end) + DiffSVGang (end) + WV (end):D (end) + WV (end):SVG (end) + WV (end):DiffVang (end) + WV (end):DiffSVGang (end) + D (end):SVG (end) + D (end):DiffVang (end) + D (end):DiffSVGang (end) + SVG (end):DiffVang (end) + SVG (end):DiffSVGang (end) + TOD (end):WV (end) + TOD (end):D (end) + TOD (end):SVG (end) + TOD (end):DiffVang (end) + TOD (end):DiffSVGang (end)
Selection dependent on starting location	WV (start):WV (end) + D (start):D (end) + SVG (start):SVG (end) + DiffVang (start):DiffVang (end) + DiffSVGang (start):DiffSVGang (end)
Movement	log(SL) + cos(TA) + log(SL):WV (start) + log(SL):D (start) + log(SL):SVG (start) + log(SL):DiffVang (start) + log(SL):DiffSVGang (start) + log(SL):Temp (start) + log(SL):TOD (start)

Model terms covered three broad questions (Table 2). Terms involving only parameters at the end of the step were to answer questions relating to habitat selection: did covariates affect selection of a step? Interaction terms between a parameter at the start and end of the step were to investigate whether the selection strength of a parameter is dependent upon where the fish began the step – for instance, will fish select deep water if they are already in deeper water? Lastly, interactions between log(step length) and other paramters are to investigate whether the magnitude of displacement varied with other parameters. For each model, the concordance statistic (a measure of model performance) was determined.

Secondly, we wanted to be able to compare and quantify the extent of individual variation in terms of effects size of parameters, as well as general trends for the studied population – did fish differ in their relationship to parameters? What were the general trends within the population? A two-step approach was used, outlined by Fieberg et al. [38], where fish are modelled individually then coefficients averaged to describe the population. In particular, we followed a similar two-step protocol to Morrison et al. [39] to obtain the population models: coefficients from the individual models were averaged per species to describe the population. Where a coefficient was not present in an individual model, the coefficient’s value was set to zero e.g. no effect. When calculating means, coefficients that were outliers were removed as we did not want extreme coefficients to change the direction or size of the mean. Standard deviation, standard error, and the coefficient of variance (standard deviation / mean) were calculated for each coefficient. In addition, the 95% confidence intervals were calculated for each mean and a one-sample t-test applied to extract p-values for the difference between the means and zero. Population model coefficients where the 95% confidence interval included zero and p-value > 0.05 were assumed to not have a cohesive effect on a population level.

The effect of parameters upon selection were visualised via calculating the relative selection strengths (RSS) and creating log-RSS plots [40, 41]. The RSS quantifies the relative probability of one location being selected over another, where the two locations differ in only one habitat parameter. Where a parameter increases by one unit, the RSS is equivalent to the exponentiate of the model coefficient for that term [41] (and thus the log-RSS is equal to the coefficient in this context). For each parameter in the models, the log-RSS for a range of values from its minimum to maximum value was calculated, by comparing to the selection of the parameter’s mean value and where all other parameters are kept constant at their mean values. Where interaction terms existed, the log-RSS was calculated for three values of the interaction term (min and max, and the value halfway between). Four generalised linear models (GLMs) were fitted for each model term present in any fish model to explore variation in coefficient value for that term with weight, fork length and total length, along with a null model. The GLMs were compared to each other via AIC to determine if weight, fork length or total length explained observed variation in coefficient size better than a null model. We did not explore the effect of fish sex upon model coefficients, due to the low number of fish that were confidently sexed and few analysed males (two male barbel and two male grayling). We also did not explore the effect of capture location upon coefficients. For barbel, this was due to all barbel being caught in the fish ladder or counting pool. For grayling, few analysed grayling were caught in the counting pool (n = 3) compared to downstream (n = 8).

## Results

### Model structures

Individually selected models varied with regards to included terms and number of terms. Number of terms in individual models ranged from six to 21. Mean number of terms per individual model was 12.5 and median was 12. In total, 33 unique terms were present in the individual models for barbel and 29 in those for grayling.

**Fig. 2:**
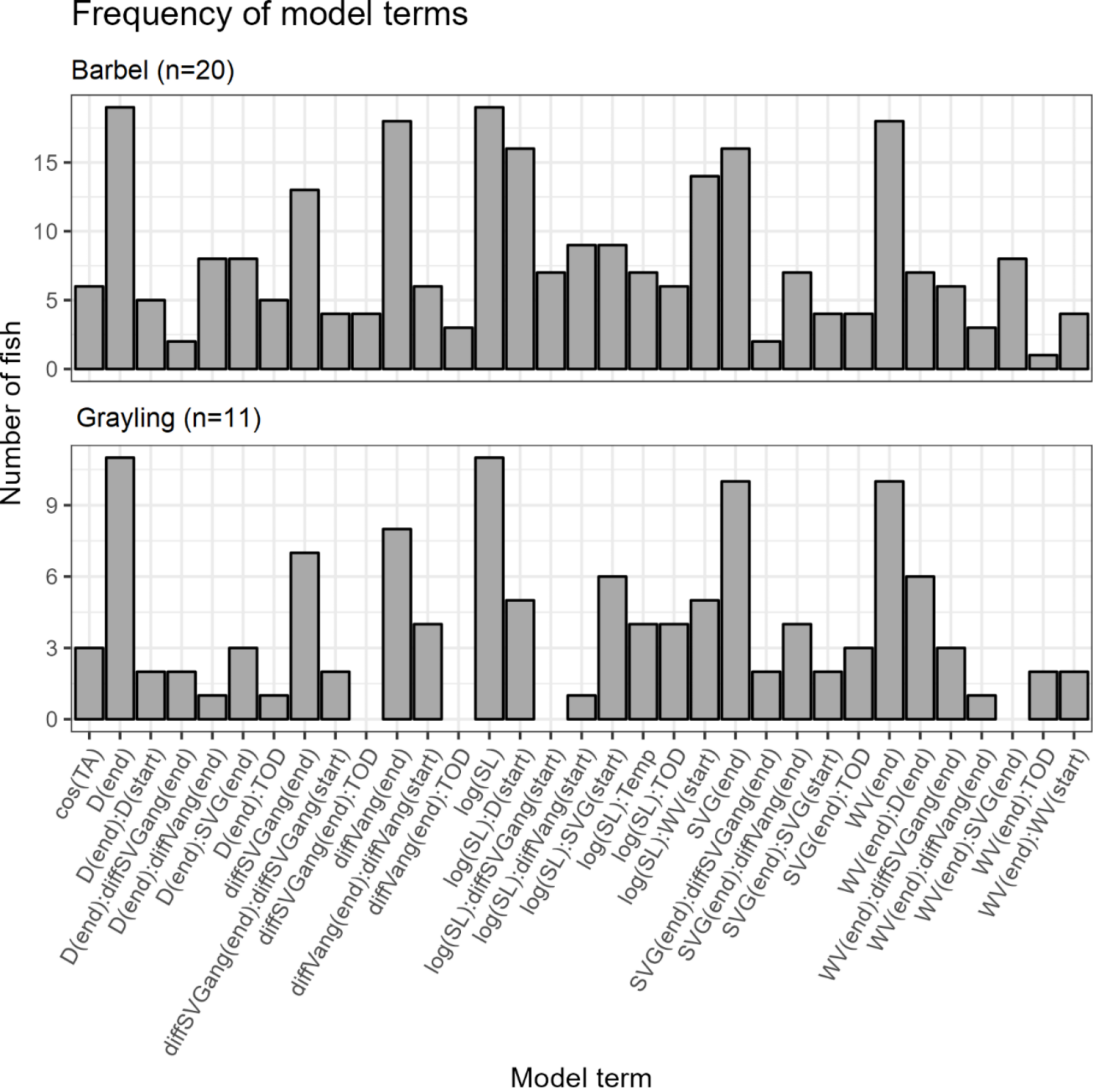
The frequency of terms in the individual models of barbel (**A**) and grayling (**B**). For clarity, parameter names have been abbreviated as defined in Table 1. (end) and (start) show if it is the parameter value at the end or start of a step. Interactions are shown by “:”

**Fig. 3:**
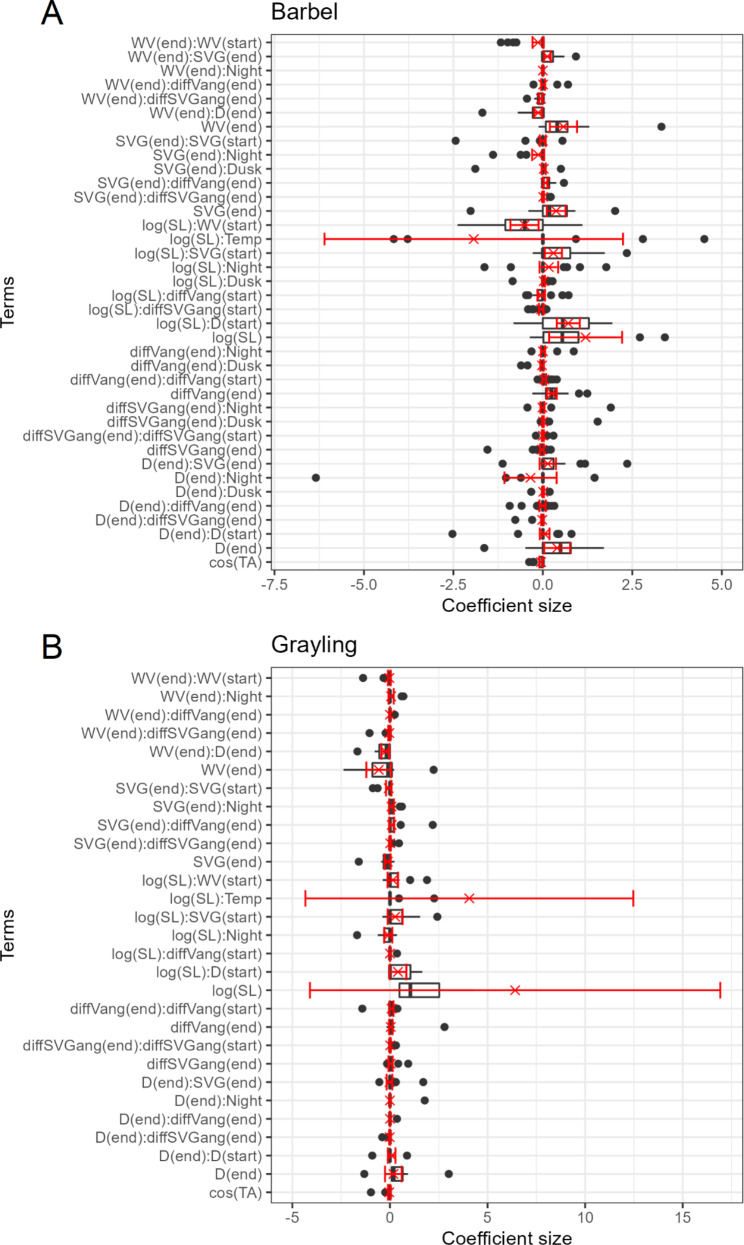
Coefficient sizes for individual barbel (**A**) and grayling (**B**) from individual models. The mean and 95% confidence intervals are show in red. Model terms are abbreviated as per Table 1 for clarity. The x-axes have been trimmed and some outliers are not shown on the plot due to their inclusion obscuring the boxes

There was a broad range of terms in models (Fig. 2). All saturated model terms were present in at least one barbel. For grayling, no individuals featured the following terms in their models: interaction between flow velocity and spatial velocity gradient (SVG) at the end of a step; interaction between the difference between fish angle and velocity angle with time of day; interaction between the difference between fish angle and SVG angle with time of day; and an interaction between the logarithm of the step length with the differences between fish angle and SVG angle. Some terms were common within and between species. For example, all grayling and all but one barbel had log(step length) and depth at the end step in their individual models. Other common terms in both species included flow velocity, SVG, the difference between fish angle and velocity angle, and the difference between fish angle and SVG angle. In addition, a number of terms describing movement via interactions with log(step length) were common: specifically with flow velocity, depth and SVG. Moreover, for several fish habitat selection and displacement varied with time of day. While common terms existed, coefficient size and direction varied (Fig. 3). Full frequency tables of terms for each species are in the supplementary file “Supplements” table S2-3.

Model performance is summarised in Table 3. Concordance of individual models varied from 0.57 to 0.81 across both species. The mean ± standard deviation of concordance for both species was 0.67 ± 0.06.

**Table 3:** Summary of performances of individual models, with the mean, maximum, minimum and standard deviation (SD) of the concordance statistics shown per species

Species	Mean ± SD model concordance	Maximum value	Minimum value
Barbel	0.67 ± 0.06	0.81	0.60
Grayling	0.67 ± 0.06	0.81	0.57

### Population trends and individual variation

**Fig. 4:**
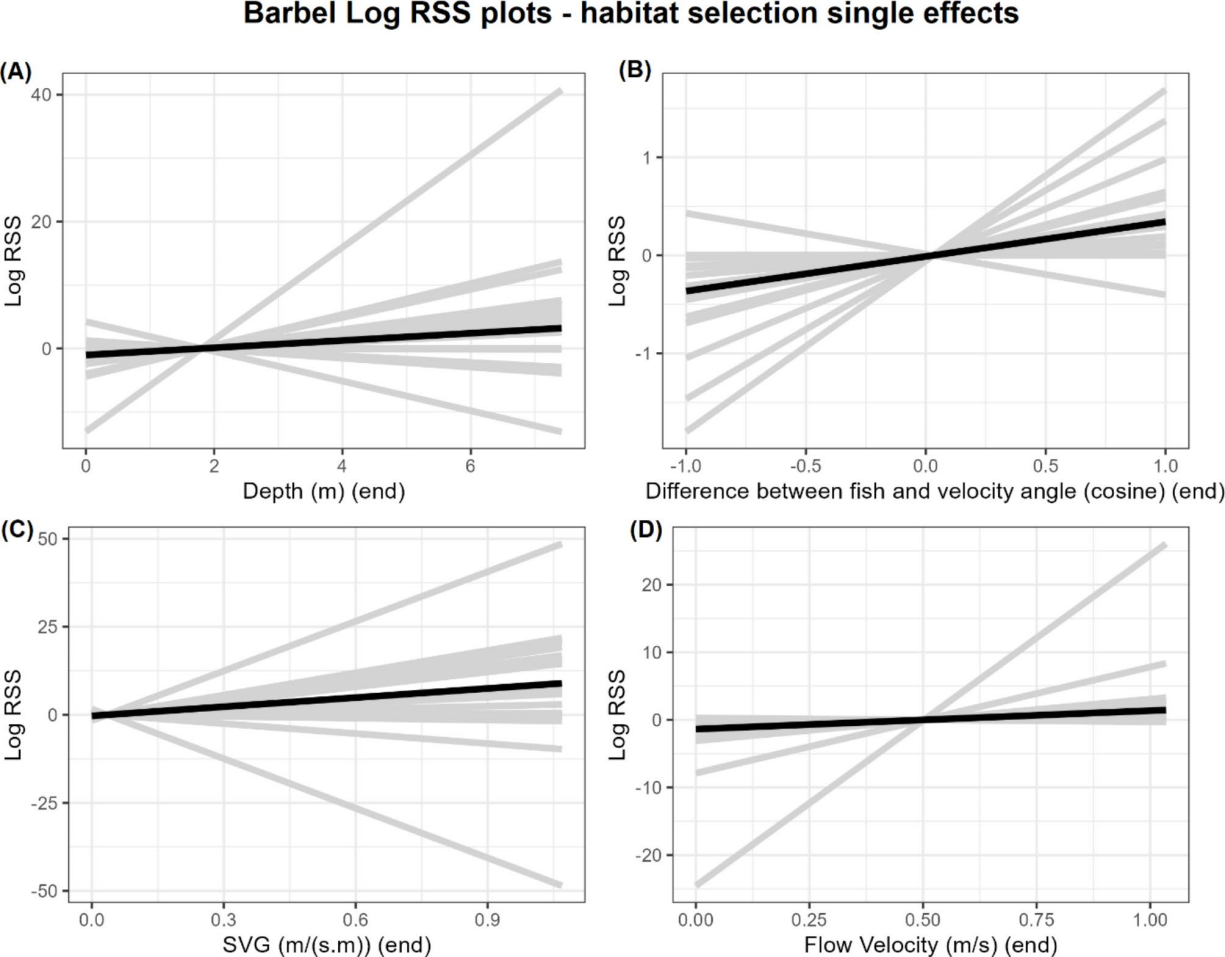
Log relative selection strength plots for single-effect habitat selection parameters for barbel. The black line shows the population effect while grey lines represent individual fish. For each parameter, the log-RSS is calculated for a range of values between the parameters maximum and minimum, while all other parameters are at their mean

**Fig. 5:**
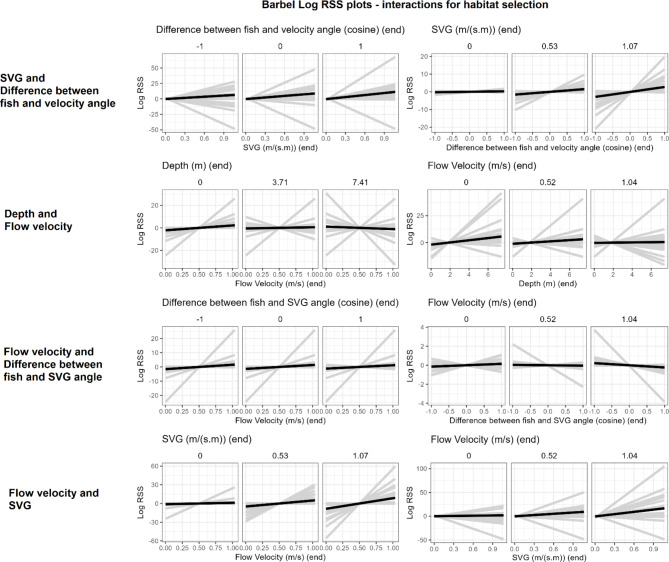
Interactions between parameters for habitat selection in barbel. Each row depicts two plots for an interaction. Per x-axis, three plots are made for three values of the interaction term. Black lines represent the mean effect size and grey lines show individual effects

**Fig. 6:**
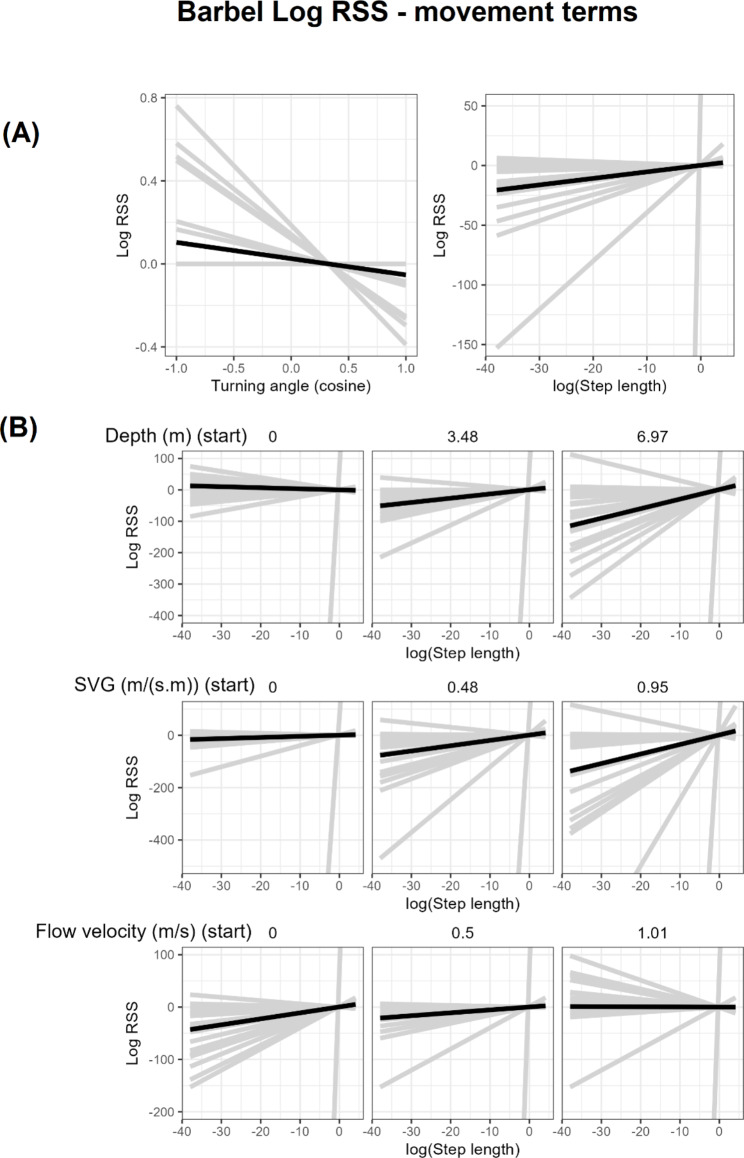
Log RSS plots for terms describing barbel movement. (**A**) Log RSS plots for the turning angle and step length. (**B**) log RSS plots showing the change in the selection strength for different step lengths at three different values of depth, SVG and flow velocity. All plots involving log(step length) have a constrained y-axis due to one fish having a coefficient that is an extreme outlier

**Fig. 7:**
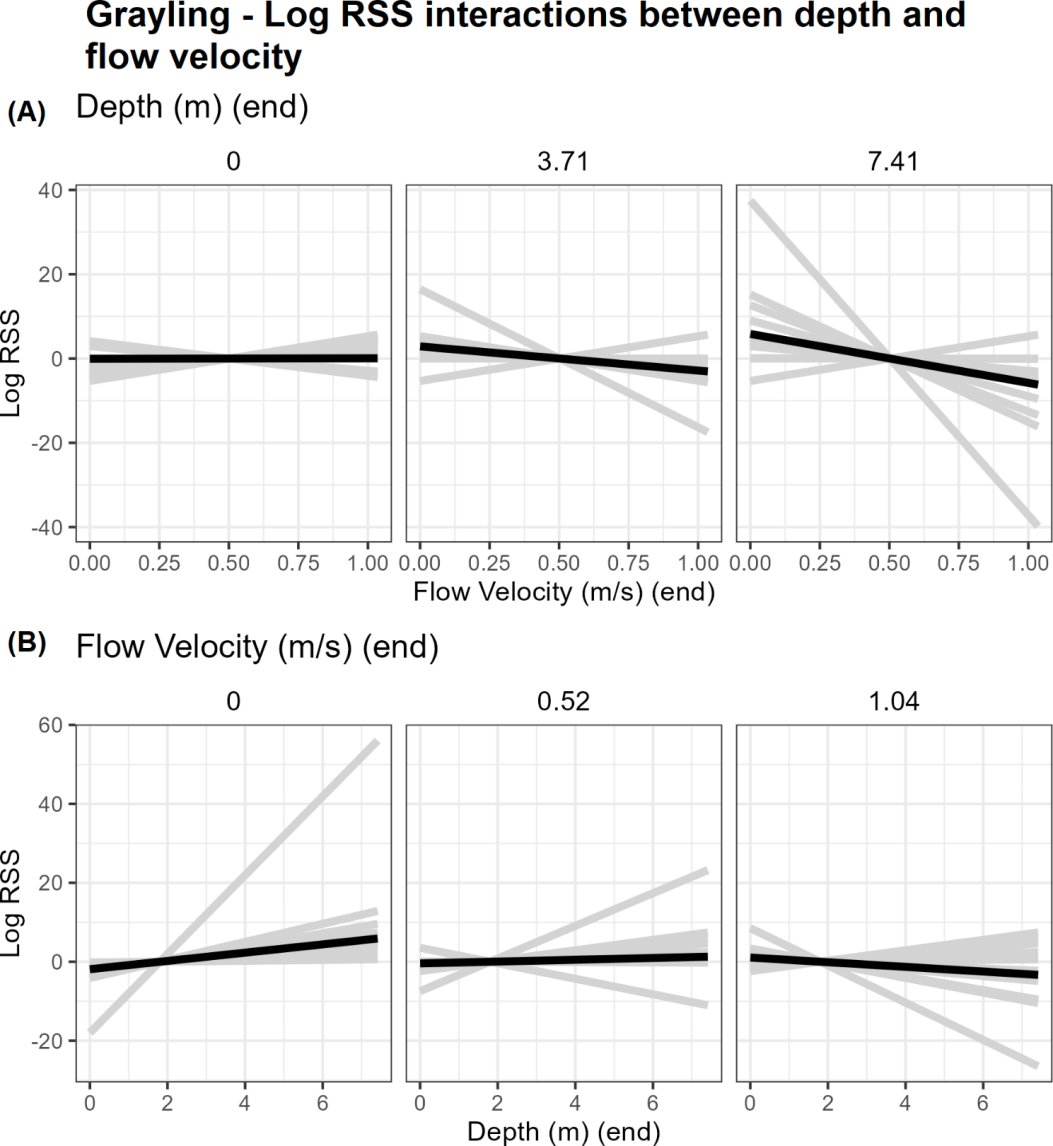
Log RSS plots for grayling, depicting the changing selection of depth and flow velocity with each other. The black line represents the mean effect while grey lines represent individuals. Figure (**A**) shows the relative selection strength of flow velocity at three different depths (minimum, maximum and the value in between). Figure (**B**) shows the changing selection strength of depth at three velocity values – maximum, minimum and halfway between

Population model coefficients are summarised in Fig. 3; Table 4. Log-RSS plots are shown in Figs. 4, 5 and 6 for barbel and Fig. 7 for grayling. Here, we only show and discuss results for terms where the 95% confidence interval of the mean does not include zero and the t-test p-value is < 0.05, e.g. the mean can be considered different to zero. Log-RSS plots for all terms are shown in the supplementary file “Supplements”, figures S1-16, and all coefficients for the population model are shown in the supplementary file “Supplements”, table S4. While interactions between time of day and other parameters were present in individual models, some individuals only had tracks during one time period. Thus, we have not included time of day interactions in our log-RSS plots. From Figs. 3, 4, 5, 6 and 7, it is evident that individual variation was high in the studied populations and standard deviations were high (Table 4).

**Table 4:** Terms from barbel and grayling models where the confidence interval of the mean coefficient does not include 0. Terms are abbreviated as per Table 1. Values are rounded to two decimal places for coefficients and to one significant figure for p values. P values are from a one-sample t-test comparing coefficients to a value of zero

Term	Barbel					Grayling				
	Mean coefficient	Standard deviation	Standard error	Coefficient of variation	P value	Mean coefficient	Standard deviation	Standard error	Coefficient of variation	P value
*Habitat selection*										
WV(end)	0.58	0.79	0.18	1.37	0.005	-	-	-	-	-
D(end)	0.40	0.75	0.17	1.88	0.03	-	-	-	-	-
SVG(end)	0.37	0.55	0.13	1.47	0.008	-	-	-	-	-
diffVang(end)	0.25	0.28	0.06	1.12	0.001	-	-	-	-	-
WV(end): diffSVGang(end)	-0.05	0.10	0.02	-1.77	0.02	-	-	-	-	-
WV(end): D(end)	-0.13	0.23	0.05	-1.78	0.02	-0.23	0.29	0.09	-1.26	0.03
WV(end): SVG(end)	0.13	0.19	0.04	1.53	0.01	-	-	-	-	
SVG(end): diffVang(end)	0.07	0.12	0.03	1.63	0.02	-	-	-	-	-
*Movement*										
log(SL)	1.19	2.13	0.49	1.78	0.02	-	-	-	-	
log(SL): D(start)	0.71	0.67	0.15	0.94	0.0002	-	-	-	-	
log(SL): WV(start)	-0.51	0.82	0.19	-1.60	0.01	-	-	-	-	
log(SL): SVG(start)	0.29	0.50	0.11	1.70	0.02	-	-	-	-	
cos(TA)	-0.05	0.10	0.02	-1.93	0.04	-	-	-	-	

#### Habitat selection of barbel and grayling

Population models indicated a wide spread of model coefficient values and thus effect on selection (Figs. 3, 4, 5 and 7). Mean coefficients, that we take to describe the general trend varied greatly. Standard deviation was large for all terms. With the exception of the interaction between depth and log(step length) for barbel, all standard deviations were greater in magnitude than the mean.

### Single effects

In general, barbel selected for faster flow velocities (Fig. 4): flow velocity had the highest mean coefficient of any habitat selection parameter (Table 4). Not all barbel selected strongly for flow velocity though: on an individual level, one barbel had a negative coefficient and two fish did not have water velocity in their individual models thus coefficient counted as zero in the population model (Fig. 4). Moreover, some individuals selected much more strongly for water velocity compared to the mean. Depth and SVG also had positive mean coefficients (Fig. 4), though individual variation was again broad and some fish had negative relationships. Fish also positively selected for the cosine of the difference between fish and flow velocity angles – e.g. selecting to move with a narrower angle between them and the flow, and move in the direction of the flow.

No grayling single effects for habitat selection had means different to zero, though on an individual level a range of coefficient magnitudes and directions existed. The confidence interval for flow velocity narrowly included zero with a near-significant p value (p = 0.07) from the t-test (supplementary file “Supplements”, table S4). The mean coefficient for flow velocity was negative, indicating a near-significant negative effect of flow velocity on grayling habitat selection.

### Interactions for habitat selection

For barbel, interactions were also included between habitat parameters. A positive interaction existed between flow velocity and SVG, and between SVG and the difference between fish and velocity angle. For each of these interactions, when the value of one parameter increased, the other parameter had a stronger positive effect on selection (Fig. 5). Negative interactions existed between flow velocity and the difference between fish and SVG angle and between flow velocity and depth, e.g. selecting shallower depths when flow is fast and selecting deeper waters when flow is slow, and vice versa (Fig. 5).

The only interaction that was different to zero in the grayling population model was between flow velocity and depth. Flow velocity and depth had a negative interaction for grayling on average, meaning at higher flow velocities, fish avoided deeper waters (Fig. 7).

#### Comparing start and end habitats

No interactions between start and end habitats had coefficients different from zero in the population models of either species, meaning preference for an environmental parameter was not affected by the environment at a previous time step.

#### Movement of barbel and grayling

There was broad individual variation in the movement terms for barbel. Barbel selected on average for larger step lengths, though effect varied greatly in size between individuals. In particular, one individual had a step length coefficient over 50 times larger than the next largest coefficient (coefficient size = 458.14, mean coefficient size = 1.19). The selected step lengths varied with water velocity, depth and SVG. In faster water, barbel on average swam slower, although this trend was not universal. Barbel swam faster when in deeper water or when SVG was greater, but again some individuals did the opposite. For barbel, there was also a negative relationship with turning angle, indicating a tendency to select for turning over straight movement.

For grayling, on a population level no coefficient means were different to zero thus displacement was not affected by habitat or time of day. Effects were present for individual fish but variation was too great to describe a population trend. A near-significant effect was present in the interaction between the logarithm of the step length and depth (p = 0.06): the coefficient here was positive so there is weak support for grayling to move faster in deeper water (supplementary file “Supplements”, table S4).

#### Relationship between fish size and model coefficients

Comparing coefficients with individual size for terms in Table 4 found no relationships for any term with size, thus habitat selection and movement did not vary with barbel size. For grayling, the term in the population model did not vary with size. Two other terms did vary significantly with grayling weight: specifically, the interaction between depth and the difference between fish and SVG angle, and between depth and the difference between fish and flow velocity angle. However, these two terms were only present in very few fish (n = 2 and n = 1 respectively), which represented the largest fish caught.

## Discussion

To date, few applications of step selection functions (SSFs) to fine-scale data or to the aquatic environment exist. Our exploratory step selection analysis applied to fine-scale tracks of migrating barbel and grayling approaching a fish pass is one of the first fine-scale applications of step selection functions to an aquatic system and provides insight into habitat selection and movement of the two study species. Fish pass approach behaviour was analysed for 31 individuals, revealing wide individual variability in the effect of parameters upon step selection. Individual variability manifested in two ways: via terms retained by individual models and via variability in coefficient size. Our results highlight the extent of individual variation and suggest an important role for habitat upon displacement. However, our analysis has focused on exploratory modelling and our model has not yet been cross-validated and will likely require changes before use for predicting habitat selection in barrier environments. We hope our results can inform and direct future studies and management.

Our study agrees with other work finding barbel to be rheophilic. Out of habitat parameters, flow velocity had the strongest effect on barbel selecting a step. On average, flow velocity had a positive effect on selection – a higher flow velocity at the end of a step increased the likelihood that a fish would select the step. Barbel are known to prefer areas of relatively faster flow [42], although not always. For example, Capra et al. [17] found most barbel individuals avoided high depth-averaged velocities. The reason for differences between our results and Capra et al.’s [17] is unclear, but may have arisen due to site-specific differences or due to the telemetry dimension. While depth and flow velocity ranges were similar, Capra et al. [17] studied barbel in a hydropeaking river and after spawning, compared to our study of migrating barbel. Moreover, both our studies tracked the 2-dimensional movement of fish: incorporating depth measurements of fish and the difference in environmental parameters throughout the water column could clarify relations drawn from 2D studies. More 3D telemetry of fish around barriers is needed. With regards to flow velocity, in our study not all barbel had a positive relationship with flow velocity. For two fish, flow velocity was not included in their individual model so is assumed to have no effect, and one fish had a negative coefficient. In addition, we found barbel to select for deeper waters. Similar findings have been reported for adult barbel [17, 43, 44]. Spatial velocity gradient (SVG) had a positive effect on selection and barbel selected for higher SVGs. Accelerating and decelerating flows have received much attention with regards to their ability to attract or deter fish and the specific effect can be dependent on other parameters [20, 45, 46]. For example, under low flow conditions, flow acceleration can attract eels [20]. With our study, a positive interaction existed between flow velocity and SVG: as flow velocity increased, the selection strength of SVG also increased. Lastly, with regards to the difference between fish and velocity angles a positive relationship existed for barbel and they were more likely to select a step if it were in the direction of the flow. As a finding, this is interesting: given our fish are migrating upstream, one would expect them to swim into the flow, e.g. select for a large angular difference (and smaller cosine). While we used a 20 s time step and the angle of a fish in a time step may not fully represent its true angle during that period (e.g. may not have moved directly between the two points), Lamonica et al. [34] showed that when interpolating a 3 s track to 30 s, the underlying movement pattern was preserved. Thus, it is likely that our 20 s time step also retains the finer-scale movement patterns.

Another consideration is tagging effects. Our results showed that barbel selected for small differences between themselves and the flow, e.g. with the flow, and could be due to tagging effects: capture and tagging could have interrupted their movement and disturbed direct upstream movement. Tagging effects upon behaviour are widely reported across different species [30, 47, 48], though the effect on habitat selection is little known. Given our fish were tagged during the spawning migration and tracks analysed soon after, it is possible that we may have tagging effects present in our fish. Moreover, most barbel were caught in the upstream counting pool of the fish ladder (e.g. had already entered and passed the ladder), and whether they had habituated to the study site or remembered the route is unknown, as fish are capable of spatial learning [49]. With regards to habituation, multiple approaches by individual fish were included in the analysis and visual examination of approaches showed fish did not take identical routes on subsequent approaches to the fish pass, yet we do not know the effect upon habitat selection. Ideally, fish should be caught and tagged in advance of the spawning migration and before entering the study site, preventing potential existing familiarity with the study site or the effects of capture and tagging upon fish behaviour. However, logistical reasons prevented tagging fish earlier (e.g. a very low catch efficiency in winter and very few barbel in counting pools). Researchers could therefore opt to tag fish in the preceding spring and select tags with sufficient battery life for detection in the subsequent spawning migration. Downsides of such a method are potential low return rates of tagged fish and thus high costs (in terms of number of tagged fish and an intensive fishing campaign to catch enough fish). Moreover, data on return rates of fish tagged in the year before tracking begins are lacking in our system, but generally a large proportion of fish may not return to the study site in subsequent years.

Our population model for grayling had only one term where the 95% confidence interval did not include zero. This was a negative interaction between flow velocity and depth. A negative interaction for this term also existed for barbel, indicating preference for shallower waters when flow velocity is faster or deeper water when flow velocity is slower, and vice versa. Barbel additionally had other interactions between habitat terms: between flow velocity and SVG; between SVG and the difference between fish angle and flow angle; and between flow velocity and the difference between fish angle and SVG angle. Interactions between hydraulic terms should be cautiously interpreted at this stage. While selection strength for one parameter changed as the other parameter increased, whether this is due to fish behaviour or a relic of hydraulic interactions is hard to distinguish. Did fish choose different steps or is it due to the changing availability of the parameters relative to each other? Habitat selection analysis is sensitive to availability [50]. While SSFs limit surveyed availability to around the fish’s movement path and correlations between parameters were low in our data, hydraulic variables are still linked. For example, the deepest parts of our study site below the HPP had slower flow velocities while higher SVG values often coincided with areas of higher flow velocity. The linkage between hydraulic terms may thus affect resulting interactions in the model – for example, we report a negative interaction between depth and flow velocity, yet the deepest parts of the study site generally had slower flows compared to elsewhere. Has this region influenced overall results? Alternatively, the changing availability could influence coefficients in a different way to expected. If values of a habitat is rarer under certain conditions, it may appear to be positively selected with low levels of use [50]. By contrast, if a value of a habitat is abundant, even high usage may have low or negative coefficients [50–52]. Limitations such as this are inherent to the study site and the statistical trends described here warrant further investigation.

We did not examine time of day on the population level. For many fish, final tracks approaching the fish pass occurred during only one time period (mostly daytime), thus we couldn’t examine differences in behaviour with time of day for many fish. On an individual level, some fish did experience variation in habitat selection and movement with time of day, for example moving faster or slower depending on the time period or selecting habitat differently. While such relationships were not significant at the population level due to lack of data, it highlights an area of interest for future study. Diel activity patterns have been reported in barbel [53] and diurnal patterns in fish passage and passage success are reported in other species [14, 54]. Moreover, the tracks used in this study were filtered: overall, fish had detections across multiple time periods but when forming our data, data from other time periods may not have been retained in the final data set. Thus, while we have not reported a significant effect of time of day at the population level, its effect on some individuals warrants future attention.

Parameters describing movement were also of importance. The logarithm of the step length was one of the most frequent parameters included in individual models and remained important on a population level for barbel. Its inclusion may reduce bias of other parameters [55, 56] but also provide insight on fish displacement. Indeed, terms involving the logarithm of the step length had larger coefficients for barbel compared to many habitat selection parameters, indicating it has a stronger effect on the selection of a step than habitat selection. When in deeper water or water with a higher SVG, fish were more likely to swim faster. By contrast, flow velocity had a negative relationship with step length. Whether our observed relationships are indicative of a behavioural decision by fish or due to other unaccounted-for variation (e.g. fish distance over ground being less when flow is faster), we do not yet know. While Gutmann et al. [57] found barbel displacement to be less during periods of higher flow, their study concerned movement on a much coarser scale than presented here.

Though only one term remained significant on the population level for grayling, in individual models many terms were selected. The variation in coefficient direction and magnitude prevents at this stage general trends to be described for grayling. Some terms in the grayling population model narrowly included zero in their confidence interval. For example, flow velocity had a 95% confidence interval of -1.21 to 0.06 while an interaction between the logarithm of the step length and depth had a 95% confidence interval of -0.02 to 0.84. Thus, there is weak evidence for an effect in grayling and with flow velocity most grayling had a negative relationship. Our wide individual variation in both barbel and grayling and subsequent loss of significant effects may be a relic of the methods. Moreover, while few terms remained significant at a population level for grayling, strong and contrasting relationships existed on the individual level and thus though we cannot generalise at this stage, future work could aim to delineate reasons behind the observed variation.

Despite the importance of many terms to individuals, wide variation and contrasting effects resulted in lack of significance on a population level. Some individuals had the opposite relationship to a parameter than the mean. For example, both SVG and depth had a mean positive effect on selection by barbel, yet some individuals reacted negatively to them. Individual variation has previously been reported in habitat selection for freshwater fish, including barbel [17]. Barbel have also been found to have a wide individual variability in home range size [57, 58]. Moreover, in our study some individuals were outliers with regards to coefficient size, further highlighting the variability of individuals to general trends. Of note is the methods. A two-step approach as we have done here results in higher variability of model coefficients than mixed modelling approaches [38] and while mixed-modelling approaches can be applied to conditional logistic regression [59], it is computationally more challenging than two-step. Thus, our variation reported here may be larger than reality. While for many terms the variation in coefficient size was very large, for some that were borderline on being removed due to confidence intervals, other approaches may have found them significant on the population level.

Moreover, our results also highlight the need to consider individual variation in management. While population trends exist, some individuals had markedly different selection, e.g. as mentioned with barbel depth and SVG preferences. Contrasting differences in individual selection have been reported in other taxa, e.g. black bears [19] and moose [18]. However, there is also need to understand why such variations may arise. We found no relationship between coefficients significant on a population level with barbel size. Two grayling terms were explained by fish weight but as the terms were only present in the individual models of the largest fish, the strength of the finding is debatable and requires future work.

Residual variation in model coefficients could also be explained by variations in fish behaviour. Studied fish may vary in behavioural modes which could have in turn affected habitat selection. Of the analysed data, some fish had tracks composed solely of directed movement while others a mix of directed movement and possible searching behaviours. Where fish spent different proportions of times in different behavioural states, individual variation in pooled model coefficients could arise. Habitat selection can vary with behaviour. On coarse scales, habitat selection can vary with behaviours such as spawning [60] but on finer scales fish may select different habitat when moving, resting or foraging. Ignoring behavioural types can lead to different models for pooled data and for specific behaviours [61], and thus if we compare coefficients of a fish for one behavioural state to a fish that used two, we may see individual variation as a result of variation in behaviour. Hidden Markov models (HMMs) can shed light upon otherwise hidden behaviours of fish [62]. Already, HMMs have been combined with SSFs [63, 64] with proposals to integrate the two methods [65], although their application to fine-scale data as presented here is novel and remains challenging. Accounting for different behavioural states in SSF analysis is an important next step for understanding fish pass approach behaviour and account for remaining variation.

From a methodological perspective, here we present one of the first applications of SSFs to fine-scale data. To date, most SSF publications deal with GPS tags, where positions are recorded on a much coarser temporal resolution (e.g. 15 min to hours apart): the scale of migrating fish in the vicinity of a fish pass is seconds and indeed acoustic devices can easily emit pings every few seconds. In Thurfjell et al.’s [24] review on SSFs, three of the 14 studies had time steps finer than an hour (15 and 30 min time steps) and even the application of SSFs to snail movement had time steps of 30 min [66]. In our study, we settled upon 20 s time steps. Firstly, given the fine-scale nature of fish passage, we wanted to study movement at on a fine-scale: the environment is highly variable and fish may be making decisions on the scale of seconds, but too fine a time step (e.g. 5 s) could have noise from random movement. Moreover, positioning errors can have a proportionally larger effect at ultra-fine time steps. Preliminary comparison of fish step lengths at different temporal resolutions to the raster resolution showed at finer time steps than 20 s (10 s and 5 s specifically), most step lengths were less than the raster resolution. Our time step interpolation of 20 s was similar to Lamonica et al.’s [34] use of 30 s, where they found a 30 s interpolation preserved the underlying pattern of raw tracks simulated at 3 s intervals which was lost at time steps over a minute. Future developments have occurred for SSFs with regards to frequency: for example, the development of time-varying SSFs where key turning points of the track are identified and steps drawn between them [67], although such an approach may limit behavioural state identification as per HMMs.

Looking forward, we hope our presented results can inform and direct future work on fish passage. Firstly, our resulting analysis of parameters could direct parameter inclusion in future SSF application or for other methods. Our current model is exploratory and has not yet been cross validated to evaluate its accuracy in predicting. Future developments of the model with regards to predictability could lead to maps of predicted usage under different discharges: with this, HPP activity could be altered to provide more favourable conditions during barbel and grayling spawning migrations. Performance of individually selected models were assessed by the concordance statistic. Across both species, the concordance statistic of individual models ranged from 0.57 to 0.81 (mean 0.67 ± 0.06 for both species) which is adequate compared to other studies – for example Stewart et al. [68] reported concordance between 0.594 and 0.647 while Rodgers et al. [69] had concordances between 0.749 and 0.910. Model concordance was higher in saturated models for all bar one fish (where the final model had concordance 4 × 10^− 16^ higher), thus by performing model selection via AIC to avoid over parameterising our individual models, concordance may have suffered. Our intention with this analysis was to explore variables behind step selection of migrating fish. Here, we analysed parameters affecting individual fish movement and their relative directions, which could direct parameter inclusion in future studies. For example, we have shown terms which commonly explain fish movement on an individual level and population-level effects and such terms could aid development of future SSFs or other modelling approaches, e.g. habitat suitability models or fuzzy logic approaches, by providing information on parameters affecting fish movement and step selection. Lastly, awareness of individual variation can inform predictive tools for management. Individual based models are a growing tool for predicting fish passage, yet may not always vary individual responses to environmental parameters [29]. While we are not yet able to delineate individual variation in habitat selection into behavioural states, we do show wide individual differences in response to several hydraulic parameters by barbel and thus incorporating variation may lead to more accurate simulations.

## Conclusion

Here we presented one of the first applications of step selection functions to migrating freshwater fish. Common terms existed in individual models but for some terms their effect cancelled out on a population level. In particular, grayling results are not yet cohesive with regards to population trends and individual variation was wide. Our results for barbel show selection for faster flow and deeper waters, along with higher spatial velocity gradients, but also the presence of interaction terms affecting selection under different conditions. Future work could examine causes behind individual variation e.g. behavioural modes to further delineate habitat selection of migrating fish.

### Electronic supplementary material

Below is the link to the electronic supplementary material.

Supplementary Material 1

## Data Availability

The datasets used and/or analysed during the current study are available from the corresponding author on reasonable request.
