# Supplementary material for: Individual variation in the habitat selection of upstream migrating fish near a barrier
Source: Mov Ecol. 2023 Aug 7;11:49. doi: 10.1186/s40462-023-00414-0 (PMC10405436; doi:10.1186/s40462-023-00414-0)

Supplements to “Individual variation in the habitat selection of upstream migrating fish near a barrier.”

Table of Contents

[Filtering tracks 1](#_Toc137820792)

[Tagged Fish Data 3](#_Toc137820793)

[Term Frequency Tables 7](#_Toc137820794)

[All Log-RSS Plots 9](#_Toc137820795)

[Coefficients of Population Models 21](#_Toc137820796)

[Environmental rasters 23](#_Toc137820797)

## Filtering tracks

Tracks were filtered to remove tracks that may not depict migration behaviour. Such tracks were defined as:

1. Tracks without any directed movement, where the fish spent the entirety of the track moving in a “searching” pattern near the pass. Our interest was in fish as they approached the pass, which could involve search behaviours (e.g. searching for the pass). However, some tracks had no directed movement to the pass – the fish spent the entirety of the track moving in the same area. Such behaviour could be non-migratory, e.g. feeding, though we cannot know for certain. We removed such tracks.
2. Similarly, some tracks ended near their start point – e.g. they started near the pass but beyond our 10m threshold. These tracks often saw the fish move greatly in the river. However, since the tracks began near where they ended, we considered that the tracks may not show migratory behaviour, e.g. that the detection within 10m of the pass may be coincidental, if the 60min track began near there.
3. Tracks where, due to filtering, the final detection was not near the pass. While initially all tracks ended at the pass, subsequent processing (specifically, interpolating with crawl and conversion from points to steps), could have resulted in data loss. For example, if the final detection pre-crawl was near the pass, but was preceded by a gap of over 1 minute, the detection would be discarded. Alternatively, if the final detection belonged to a segment 10> detections, crawl would also not be applied and the segment discarded. Finally, when converting to steps, a collection of steps (termed, burst) were only retained if >3 steps were in a burst, as that is required for calculating a turning angle. As a result, it was possible for some tracks to no longer approach the pass and such tracks were removed.

To meet point 1, 5 of the co-authors met and classed a subset of tracks as “good” or “bad. For each track, various attributes were calculated for each step/end detection, such as: straightness index (for 1, 5 and 10min windows), to describe the straightness of the path; net-square displacement; and total distance travelled in a track. Max, min, mean and standard error values were calculated for each attribute. The resulting information was fed into a decision tree to identify if any parameters could separate good and bad tracks. The decision tree indicated that filtering for tracks where the maximum net-square displacement was greater than 40 would be a good threshold.

Final thresholds used to remove tracks were:

1. Max net-square displacement for a track less than 40
2. The first detection occurred within 15m of the pass or the first and last detection were within 15m of each other.
3. The final point was more than 20m away from the pass. The threshold of 20m was chosen as, while it exceeds the initial 10m threshold, we accept that some data may still be lost due to previous data processing. With the 20m threshold, we know that the fish still (soon after) came within 10m of the pass and the remainder of the track could still provide insight into the habitat selection as they approached.

After the above, tracks were visually inspected. One bad track remained – while this track started away from the ladder and ended near it, it was missing detections for most of the approach to the ladder. There were a series of detections far from the ladder, and then a cluster of detections near the ladder entrance. As we were thus missing data as the fish approached the pass, this track was removed.

## Tagged Fish Data

*Table S1. Data on fish caught and tagged in this study. The number of pass approach tracks shows the number of remaining tracks showing the fish approaching the fish pass that formed the analysed data. Fish where the number of pass approach tracks is zero were therefore not analysed in this study.*

| Tag_ID | Species | Tagging date | Catch method & location description | Catch location coordinates | Release location coordinates | Fork length (mm) | Total length (mm) | Weight (g) | Sex | Number of pass approach tracks |
| --- | --- | --- | --- | --- | --- | --- | --- | --- | --- | --- |
| 46838 | Barbus barbus | 29/05/2018 | Counting pool Altusried | 47.82, 10.23 | 47.82, 10.22 | 524 | 570 | 2160 | Female | 4 |
| 46839 | Barbus barbus | 29/05/2018 | Counting pool Altusried | 47.82, 10.23 | 47.82, 10.22 | 559 | 594 | 2161.7 | Female | 0 |
| 46840 | Barbus barbus | 29/05/2018 | Counting pool Altusried | 47.82, 10.23 | 47.82, 10.22 | 420 | 456 | 932.6 | - | 1 |
| 46844 | Barbus barbus | 24/05/2018 | Counting pool Altusried | 47.82, 10.23 | 47.82, 10.22 | 469 | 504 | 1285.8 | - | 3 |
| 46845 | Barbus barbus | 24/05/2018 | Counting pool Altusried | 47.82, 10.23 | 47.82, 10.22 | 449 | 496 | 1106.8 | - | 1 |
| 46846 | Barbus barbus | 24/05/2018 | Counting pool Altusried | 47.82, 10.23 | 47.82, 10.22 | 424 | 460 | 911.2 | - | 1 |
| 46847 | Barbus barbus | 24/05/2018 | Counting pool Altusried | 47.82, 10.23 | 47.82, 10.22 | 554 | 619 | 2343.3 | - | 1 |
| 46848 | Barbus barbus | 24/05/2018 | Counting pool Altusried | 47.82, 10.23 | 47.82, 10.22 | 511 | 545 | 1567.6 | - | 3 |
| 46849 | Barbus barbus | 29/05/2018 | Counting pool Altusried | 47.82, 10.23 | 47.82, 10.22 | 449 | 480 | 1180.5 | Female | 1 |
| 46850 | Barbus barbus | 24/05/2018 | Counting pool Altusried | 47.82, 10.23 | 47.82, 10.22 | 489 | 526 | 1465.7 | Female | 4 |
| 46851 | Barbus barbus | 24/05/2018 | Counting pool Altusried | 47.82, 10.23 | 47.82, 10.22 | 302 | 330 | 327.2 | - | 2 |
| 46852 | Barbus barbus | 24/05/2018 | E-fishing, in the fish ladder | NA | 47.82, 10.22 | 424 | 460 | 1053.2 | - | 8 |
| 46853 | Barbus barbus | 24/05/2018 | Counting pool Altusried | 47.82, 10.23 | 47.82, 10.22 | 427 | 461 | 1010.6 | - | 2 |
| 46854 | Barbus barbus | 24/05/2018 | Counting pool Altusried | 47.82, 10.23 | 47.82, 10.22 | 534 | 585 | 2105.1 | - | 4 |
| 46855 | Barbus barbus | 24/05/2018 | Counting pool Altusried | 47.82, 10.23 | 47.82, 10.22 | 544 | 594 | 2212.7 | - | 1 |
| 46856 | Barbus barbus | 24/05/2018 | Counting pool Altusried | 47.82, 10.23 | 47.82, 10.22 | 481 | 513 | 1544.1 | Female | 1 |
| 46857 | Barbus barbus | 24/05/2018 | E-fishing, in the fish ladder | NA | 47.82, 10.22 | 421 | 457 | 822.9 | - | 3 |
| 46858 | Barbus barbus | 17/05/2018 | Counting pool Altusried | 47.82, 10.23 | 47.82, 10.22 | 479 | 516 | 1306.5 | Female | 8 |
| 46859 | Barbus barbus | 17/05/2018 | Counting pool Altusried | 47.82, 10.23 | 47.82, 10.22 | 360 | 387 | 648.4 | Male | 5 |
| 46860 | Barbus barbus | 17/05/2018 | Counting pool Altusried | 47.82, 10.23 | 47.82, 10.22 | 515 | 545 | 2074.4 | Male | 9 |
| 46861 | Barbus barbus | 17/05/2018 | Counting pool Altusried | 47.82, 10.23 | 47.82, 10.22 | 428 | 466 | 1009.6 | Female | 3 |
| 46862 | Barbus barbus | 17/05/2018 | Counting pool Altusried | 47.82, 10.23 | 47.82, 10.22 | 370 | 400 | 621.2 | - | 0 |
| 46863 | Thymallus thymallus | 11/04/2018 | E-fishing, downstream HPP | NA | 47.82, 10.22 | 375 | 403 | 673.3 | Female | 0 |
| 46864 | Thymallus thymallus | 11/04/2018 | Counting pool Altusried | 47.82, 10.23 | 47.82, 10.22 | 381 | 409 | 623.2 | - | 0 |
| 46865 | Thymallus thymallus | 11/04/2018 | NA | NA | 47.82, 10.22 | 324 | 350 | 315 | - | 0 |
| 46866 | Thymallus thymallus | 11/04/2018 | Counting pool Altusried | 47.82, 10.23 | 47.82, 10.22 | 319 | 326 | 262.1 | - | 1 |
| 46867 | Thymallus thymallus | 11/04/2018 | Counting pool Altusried | 47.82, 10.23 | 47.82, 10.22 | 411 | 439 | 755.4 | - | 0 |
| 46868 | Thymallus thymallus | 04/04/2018 | E-fishing, downstream HPP | NA | 47.82, 10.22 | 398 | 420 | 791.3 | Female | 4 |
| 46869 | Thymallus thymallus | 11/04/2018 | Counting pool Altusried | 47.82, 10.23 | 47.82, 10.22 | 473 | 508 | 1250.8 | Male | 1 |
| 46870 | Thymallus thymallus | 11/04/2018 | Counting pool Altusried | 47.82, 10.23 | 47.82, 10.22 | 394 | 426 | 756.8 | Female | 0 |
| 46871 | Thymallus thymallus | 11/04/2018 | E-fishing, downstream HPP | NA | 47.82, 10.22 | 389 | 416 | 843.9 | Female | 0 |
| 46872 | Thymallus thymallus | 11/04/2018 | Counting pool Altusried | 47.82, 10.23 | 47.82, 10.22 | 394 | 411 | 640.1 | Female | 1 |
| 46874 | Thymallus thymallus | 04/04/2018 | E-fishing, downstream HPP | NA | 47.82, 10.22 | 326 | 348 | 402.1 | - | 0 |
| 46901 | Thymallus thymallus | 04/04/2018 | E-fishing, downstream HPP | NA | 47.82, 10.22 | 374 | 383 | 657.7 | Female | 4 |
| 46902 | Thymallus thymallus | 04/04/2018 | E-fishing, downstream HPP | NA | 47.82, 10.22 | 404 | 431 | 712.3 | - | 0 |
| 46903 | Thymallus thymallus | 04/04/2018 | Counting pool Altusried | 47.82, 10.23 | 47.82, 10.22 | 350 | 371 | 444.6 | - | 0 |
| 46904 | Thymallus thymallus | 04/04/2018 | E-fishing, downstream HPP | NA | 47.82, 10.22 | 467 | 494 | 1048.5 | Male | 0 |
| 46905 | Thymallus thymallus | 04/04/2018 | Counting pool Altusried | 47.82, 10.23 | 47.82, 10.22 | 413 | 425 | 642.4 | - | 0 |
| 46906 | Thymallus thymallus | 04/04/2018 | E-fishing, downstream HPP | NA | 47.82, 10.22 | 480 | 498 | 1152.9 | - | 5 |
| 46907 | Thymallus thymallus | 04/04/2018 | Counting pool Altusried | 47.82, 10.23 | 47.82, 10.22 | 309 | 331 | 317.3 | - | 0 |
| 46908 | Thymallus thymallus | 04/04/2018 | E-fishing, downstream HPP | NA | 47.82, 10.22 | 381 | 400 | 683 | - | 1 |
| 46909 | Thymallus thymallus | 04/04/2018 | E-fishing, downstream HPP | NA | 47.82, 10.22 | 373 | 389 | 634.2 | Female | 1 |
| 46910 | Thymallus thymallus | 04/04/2018 | E-fishing, downstream HPP | NA | 47.82, 10.22 | 389 | 406 | 707.3 | Male | 1 |
| 46911 | Thymallus thymallus | 04/04/2018 | E-fishing, downstream HPP | NA | 47.82, 10.22 | 299 | 309 | 233.4 | - | 0 |
| 46912 | Thymallus thymallus | 04/04/2018 | Counting pool Altusried | 47.82, 10.23 | 47.82, 10.22 | 395 | 407 | 606.4 | Male | 0 |
| 46913 | Thymallus thymallus | 28/03/2018 | E-fishing, beneath fish ladder entrance | NA | 47.82, 10.22 | 301 | 318 | 312 | - | 1 |
| 46914 | Thymallus thymallus | 28/03/2018 | E-fishing, beneath fish ladder entrance | NA | 47.82, 10.22 | 286 | 304 | 286 | - | 2 |

## Term Frequency Tables

*Table S2. Frequency of model terms in barbel individual models. Model terms are abbreviated as per table 1. Terms are shown in order of most to least frequent.*

| Species | Model term | Frequency | Percent of models with term |
| --- | --- | --- | --- |
| Barbel | D(end) | 19 | 95 |
| Barbel | log(SL) | 19 | 95 |
| Barbel | diffVang(end) | 18 | 90 |
| Barbel | WV(end) | 18 | 90 |
| Barbel | log(SL):D(start) | 16 | 80 |
| Barbel | SVG(end) | 16 | 80 |
| Barbel | log(SL):WV(start) | 14 | 70 |
| Barbel | diffSVGang(end) | 13 | 65 |
| Barbel | log(SL):diffVang(start) | 9 | 45 |
| Barbel | log(SL):SVG(start) | 9 | 45 |
| Barbel | D(end):diffVang(end) | 8 | 40 |
| Barbel | D(end):SVG(end) | 8 | 40 |
| Barbel | WV(end):SVG(end) | 8 | 40 |
| Barbel | log(SL):diffSVGang(start) | 7 | 35 |
| Barbel | log(SL):Temp | 7 | 35 |
| Barbel | SVG(end):diffVang(end) | 7 | 35 |
| Barbel | WV(end):D(end) | 7 | 35 |
| Barbel | cos(TA) | 6 | 30 |
| Barbel | diffVang(end):diffVang(start) | 6 | 30 |
| Barbel | log(SL):TOD | 6 | 30 |
| Barbel | WV(end):diffSVGang(end) | 6 | 30 |
| Barbel | D(end):D(start) | 5 | 25 |
| Barbel | D(end):TOD | 5 | 25 |
| Barbel | diffSVGang(end):diffSVGang(start) | 4 | 20 |
| Barbel | diffSVGang(end):TOD | 4 | 20 |
| Barbel | SVG(end):SVG(start) | 4 | 20 |
| Barbel | SVG(end):TOD | 4 | 20 |
| Barbel | WV(end):WV(start) | 4 | 20 |
| Barbel | diffVang(end):TOD | 3 | 15 |
| Barbel | WV(end):diffVang(end) | 3 | 15 |
| Barbel | D(end):diffSVGang(end) | 2 | 10 |
| Barbel | SVG(end):diffSVGang(end) | 2 | 10 |
| Barbel | WV(end):TOD | 1 | 5 |

*Table S3. Frequency of model terms in grayling individual models. Model terms are abbreviated as per table 1. Terms are shown in order of most to least frequent.*

| Species | Model term | Frequency | Percent of models with term |
| --- | --- | --- | --- |
| Grayling | D(end) | 11 | 100 |
| Grayling | log(SL) | 11 | 100 |
| Grayling | SVG(end) | 10 | 90.90909091 |
| Grayling | WV(end) | 10 | 90.90909091 |
| Grayling | diffVang(end) | 8 | 72.72727273 |
| Grayling | diffSVGang(end) | 7 | 63.63636364 |
| Grayling | log(SL):SVG(start) | 6 | 54.54545455 |
| Grayling | WV(end):D(end) | 6 | 54.54545455 |
| Grayling | log(SL):D(start) | 5 | 45.45454545 |
| Grayling | log(SL):WV(start) | 5 | 45.45454545 |
| Grayling | diffVang(end):diffVang(start) | 4 | 36.36363636 |
| Grayling | log(SL):Temp | 4 | 36.36363636 |
| Grayling | log(SL):TOD | 4 | 36.36363636 |
| Grayling | SVG(end):diffVang(end) | 4 | 36.36363636 |
| Grayling | cos(TA) | 3 | 27.27272727 |
| Grayling | D(end):SVG(end) | 3 | 27.27272727 |
| Grayling | SVG(end):TOD | 3 | 27.27272727 |
| Grayling | WV(end):diffSVGang(end) | 3 | 27.27272727 |
| Grayling | D(end):D(start) | 2 | 18.18181818 |
| Grayling | D(end):diffSVGang(end) | 2 | 18.18181818 |
| Grayling | diffSVGang(end):diffSVGang(start) | 2 | 18.18181818 |
| Grayling | SVG(end):diffSVGang(end) | 2 | 18.18181818 |
| Grayling | SVG(end):SVG(start) | 2 | 18.18181818 |
| Grayling | WV(end):TOD | 2 | 18.18181818 |
| Grayling | WV(end):WV(start) | 2 | 18.18181818 |
| Grayling | D(end):diffVang(end) | 1 | 9.090909091 |
| Grayling | D(end):TOD | 1 | 9.090909091 |
| Grayling | log(SL):diffVang(start) | 1 | 9.090909091 |
| Grayling | WV(end):diffVang(end) | 1 | 9.090909091 |

## All Log-RSS Plots


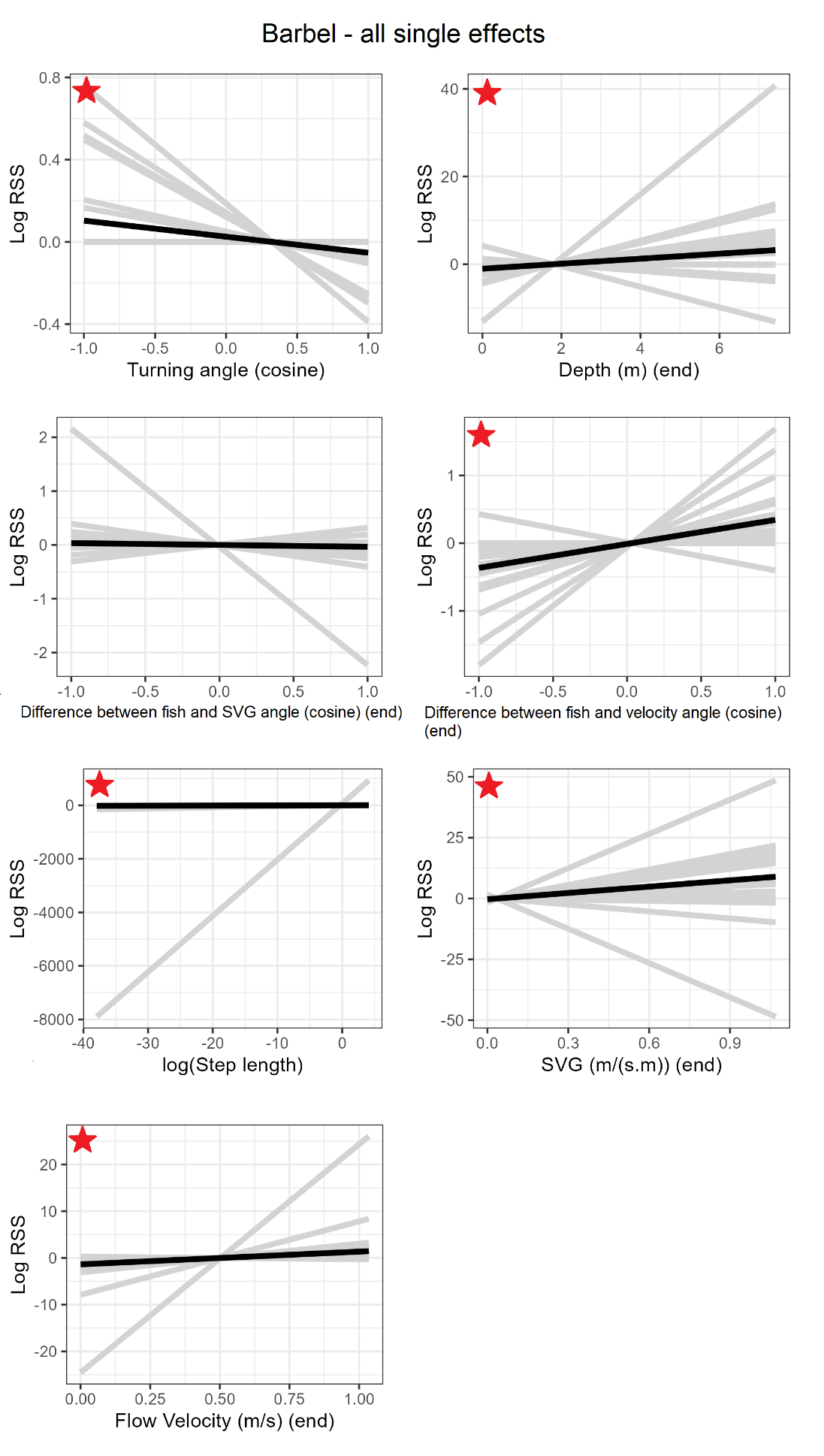


*Figure S1. All single effect log-RSS plots for barbel, labelled appropriately. Here, the black line represents the mean value and grey lines show individual relationships. Plots where the mean value is different to zero are marked by a red star.*

*
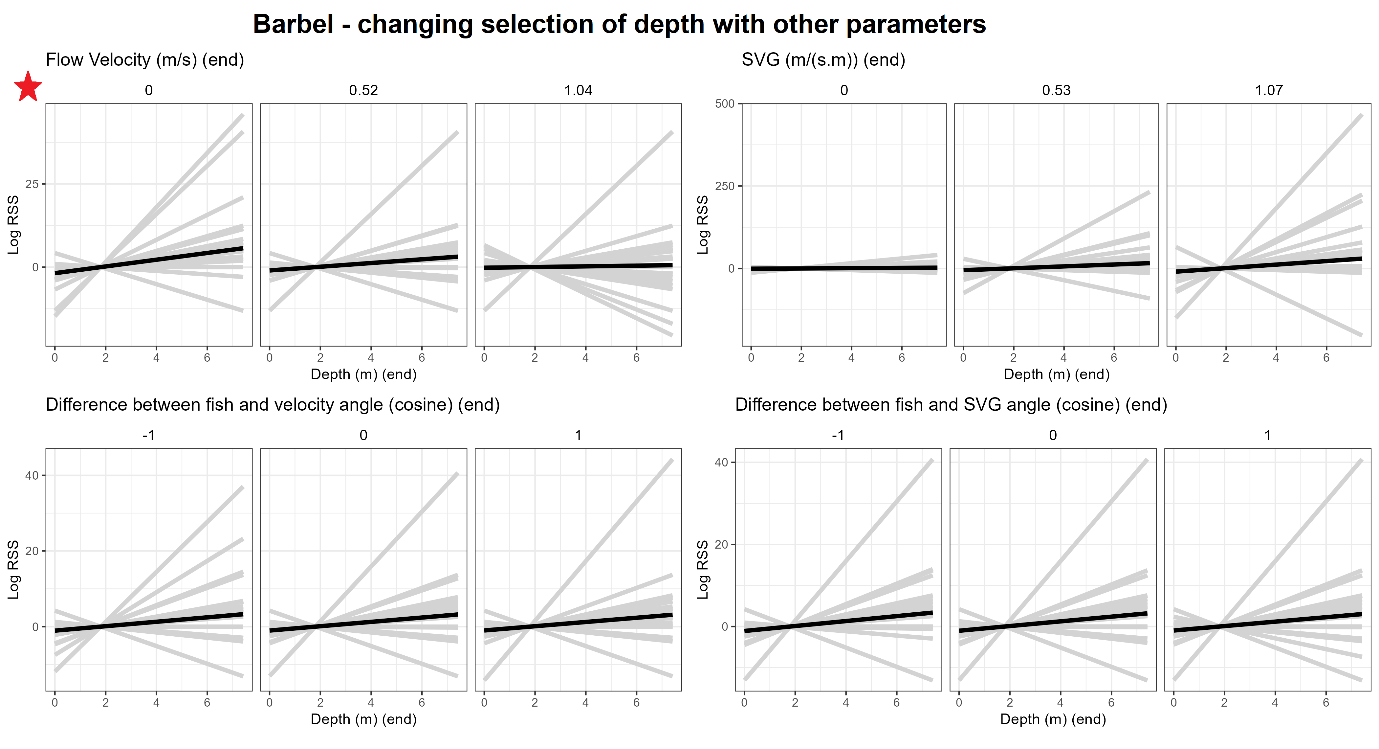
*

*Figure S2. Changing selection of depth with interacting parameters for barbel. Here, the black line represents the mean value and grey lines show individual relationships. Plots where the mean value is different to zero are marked by a red star.
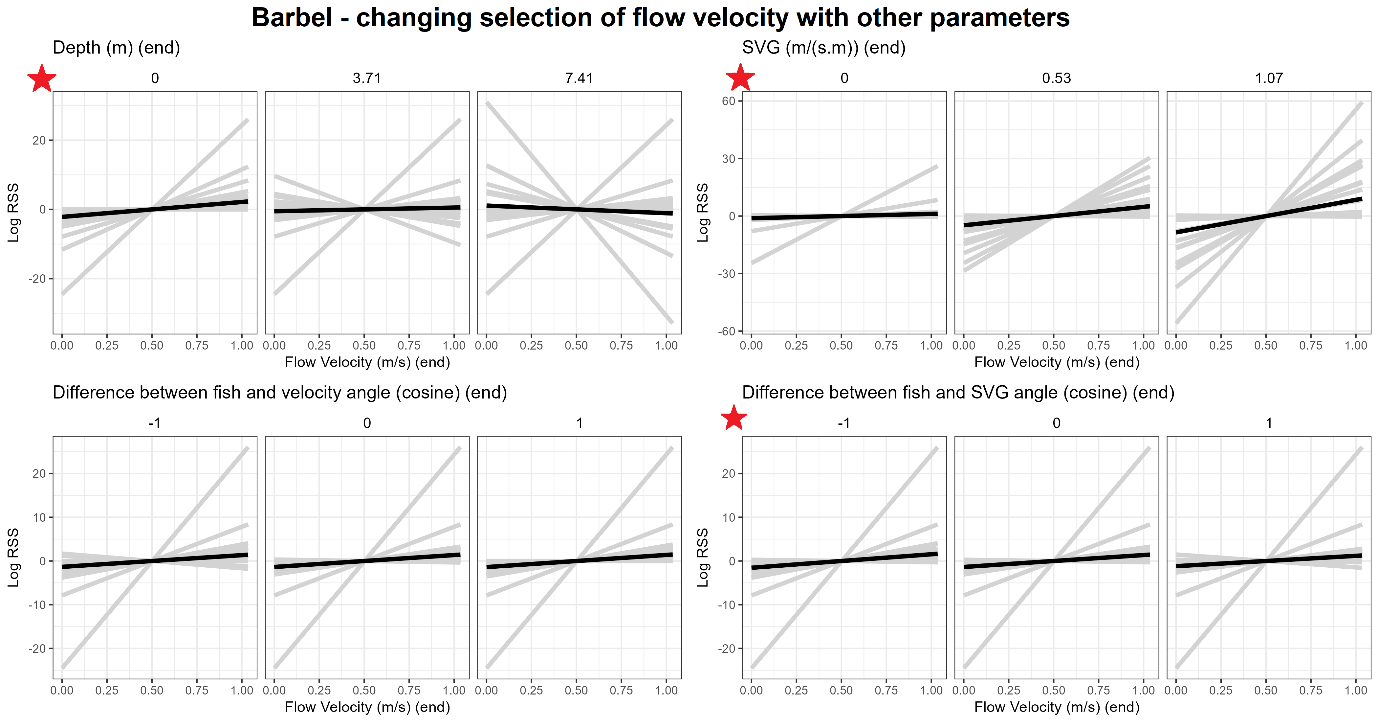
*

*Figure S3. Changing selection of flow velocity with interacting parameters for barbel. Here, the black line represents the mean value and grey lines show individual relationships. Plots where the mean value is different to zero are marked by a red star.*

*
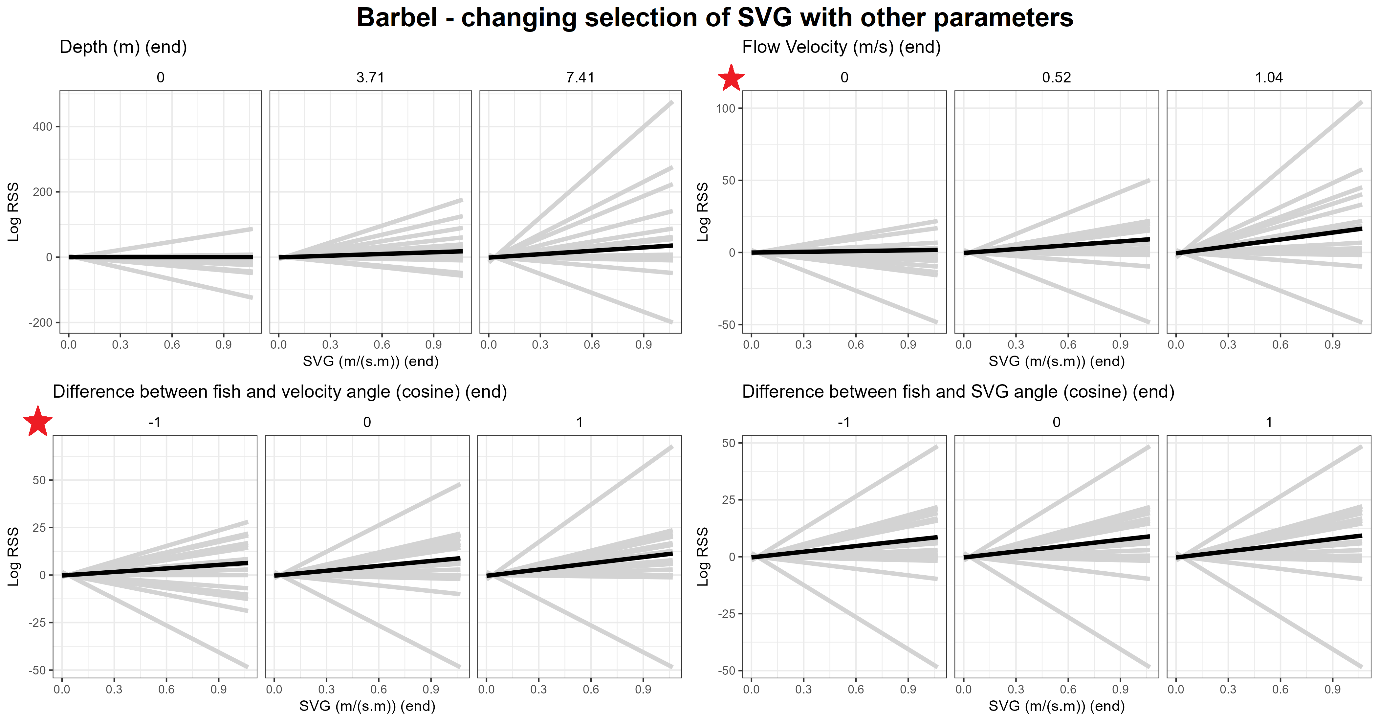
*

*Figure S4. Changing selection of SVG with interacting parameters for barbel. Here, the black line represents the mean value and grey lines show individual relationships. Plots where the mean value is different to zero are marked by a red star.
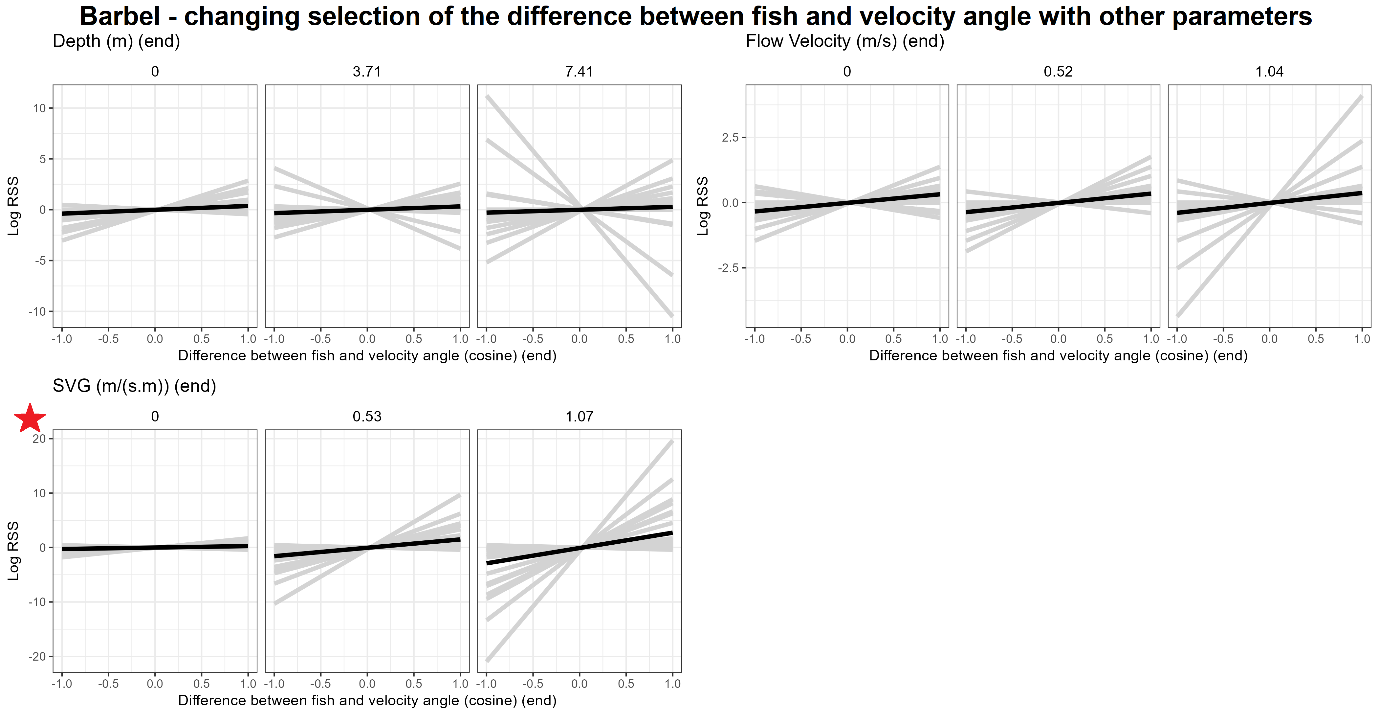
*

*Figure S5. Changing selection of the difference between fish and velocity angle with interacting parameters for barbel. Here, the black line represents the mean value and grey lines show individual relationships. Plots where the mean value is different to zero are marked by a red star.*

*
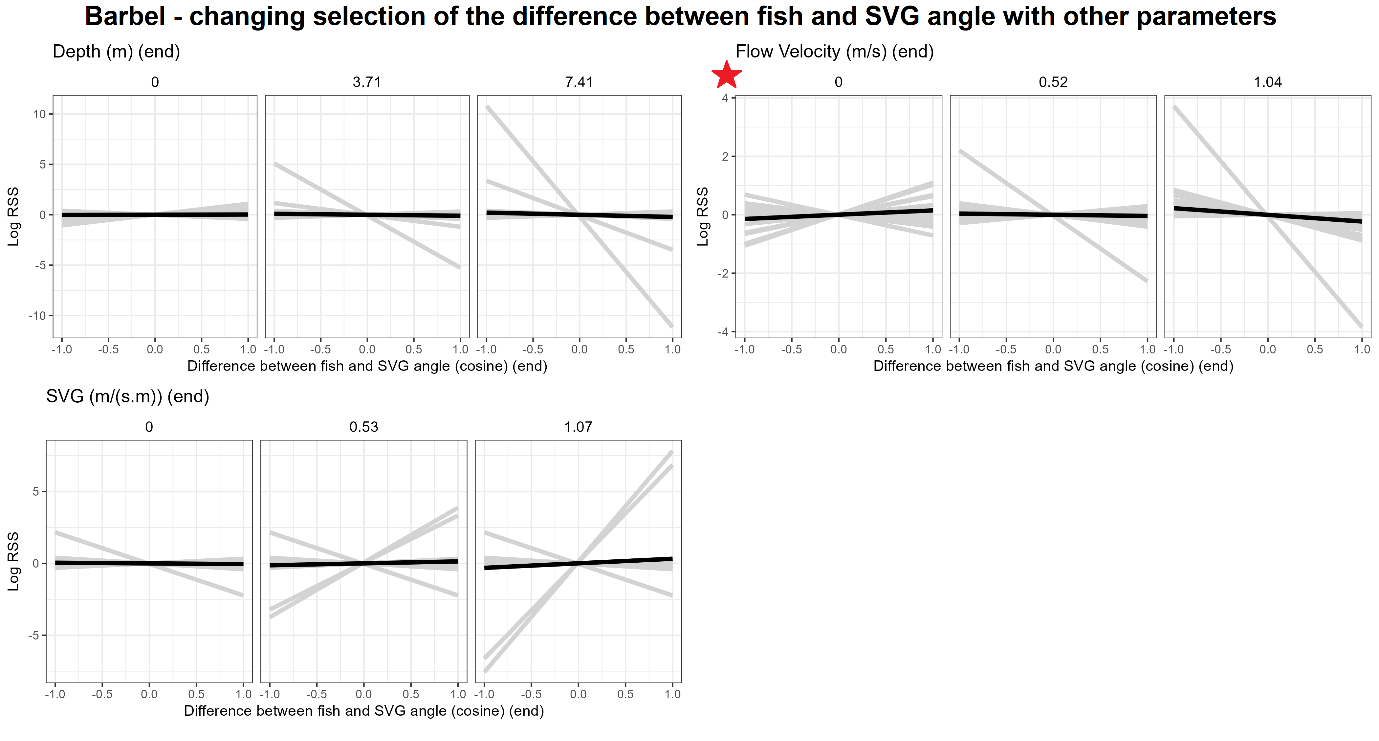
*

*Figure S6. Changing selection of the difference between fish and SVG angle with interacting parameters for barbel. Here, the black line represents the mean value and grey lines show individual relationships. Plots where the mean value is different to zero are marked by a red star.*


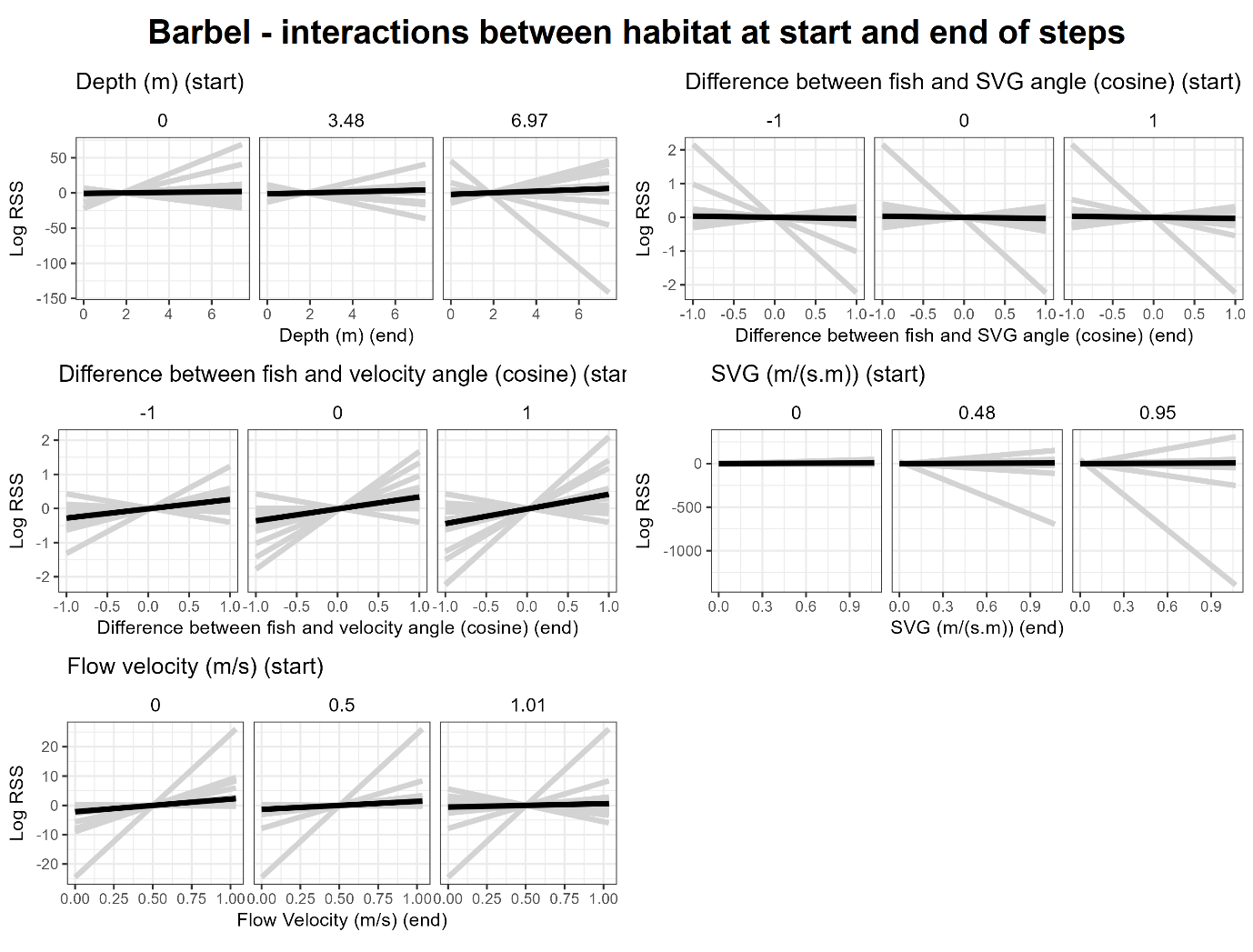


*Figure S7. All interactions between habitat at the start and end of a step for barbel. Here, the black line represents the mean value and grey lines show individual relationships. None of the means were significantly different from zero.*


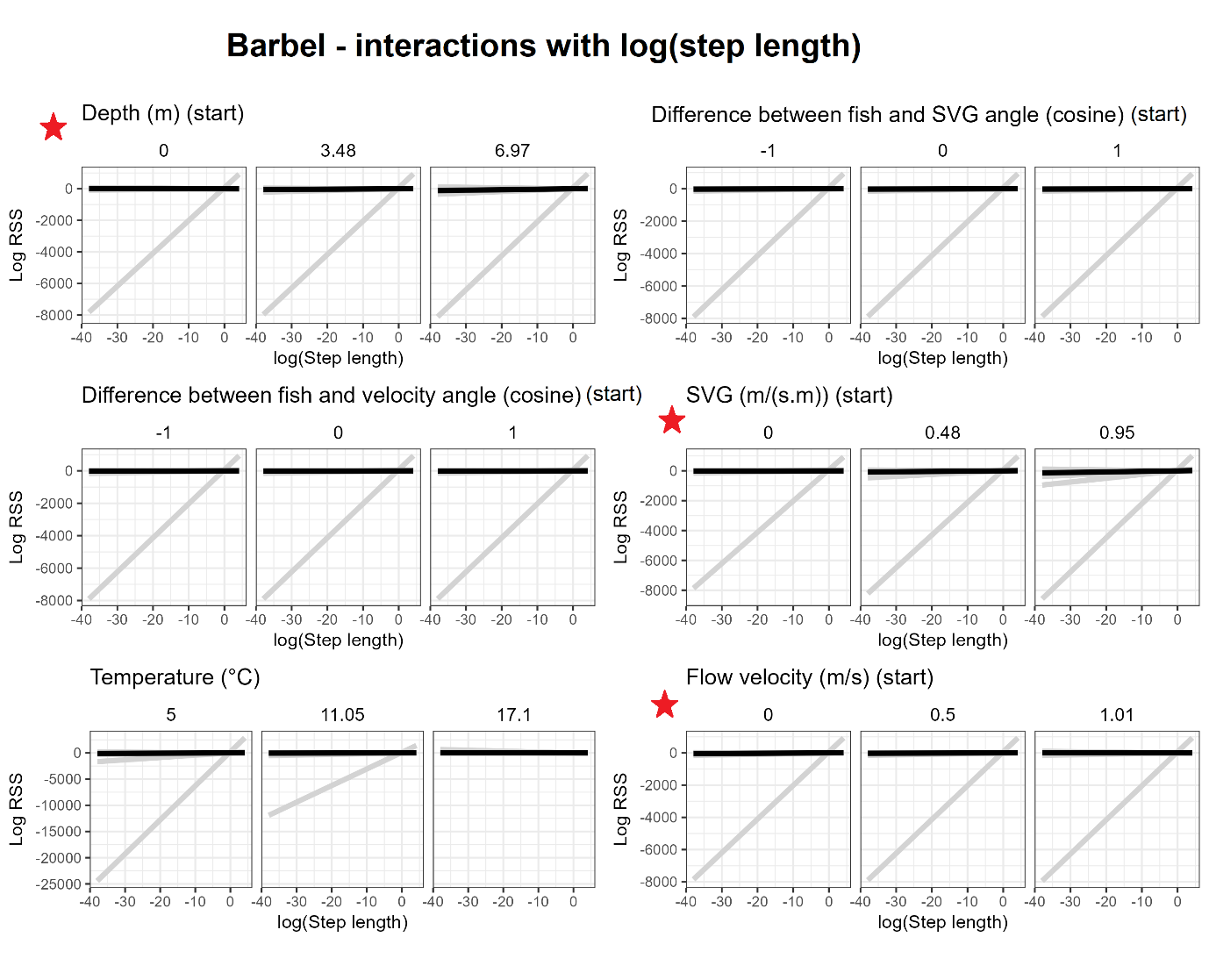


*Figure S8. All interactions between log(step length) and other parameters for barbel. Here, the black line represents the mean value and grey lines show individual relationships. Plots where the mean value is different to zero are marked by a red star.*

*
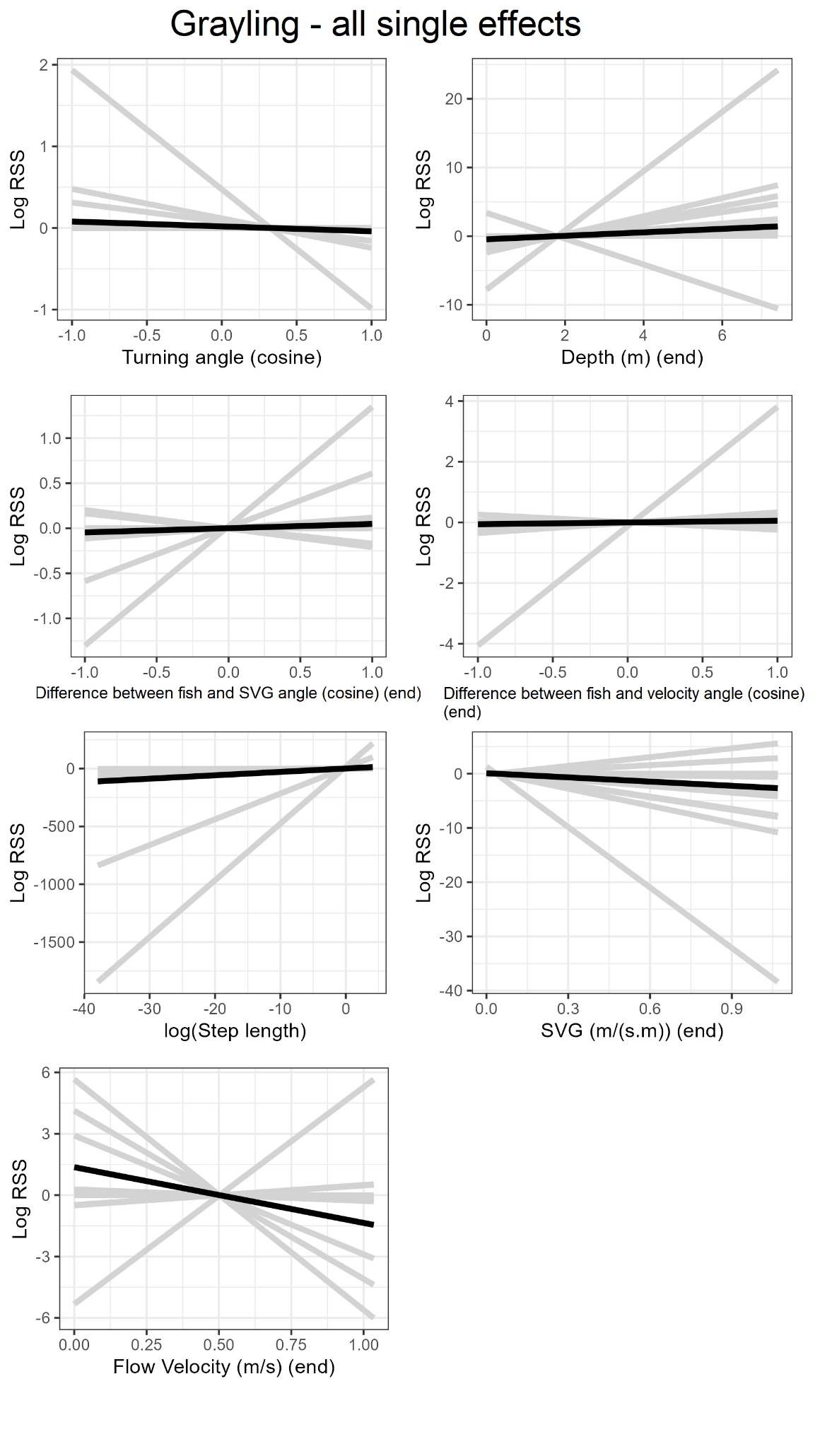
*

*Figure S9. All single effect log-RSS plots for grayling, labelled appropriately. Here, the black line represents the mean value and grey lines show individual relationships. No means were different to zero.*

*
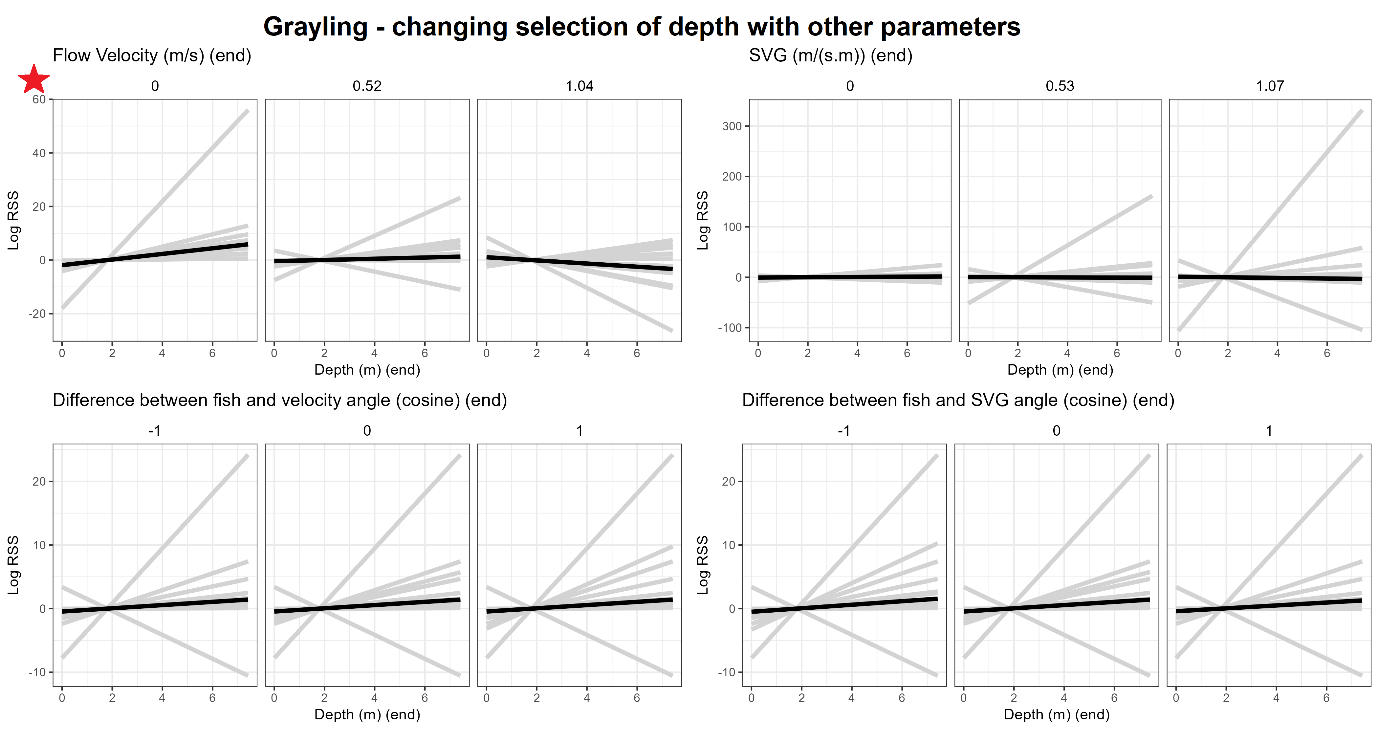
*

*Figure S10. Changing selection of depth with interacting parameters for grayling. Here, the black line represents the mean value and grey lines show individual relationships. Plots where the mean value is different to zero are marked by a red star.*

*
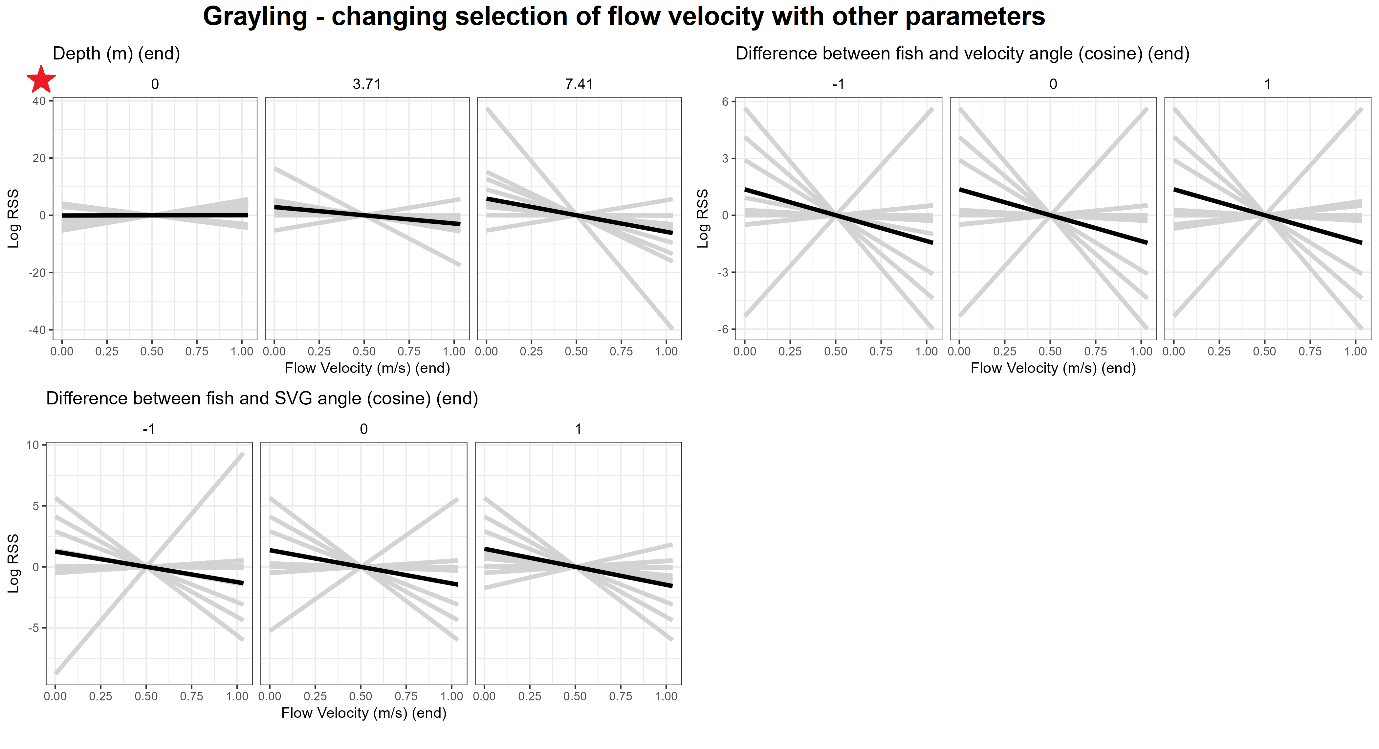
*

*Figure S11. Changing selection of flow velocity with interacting parameters for grayling. Here, the black line represents the mean value and grey lines show individual relationships. Plots where the mean value is different to zero are marked by a red star.*

*
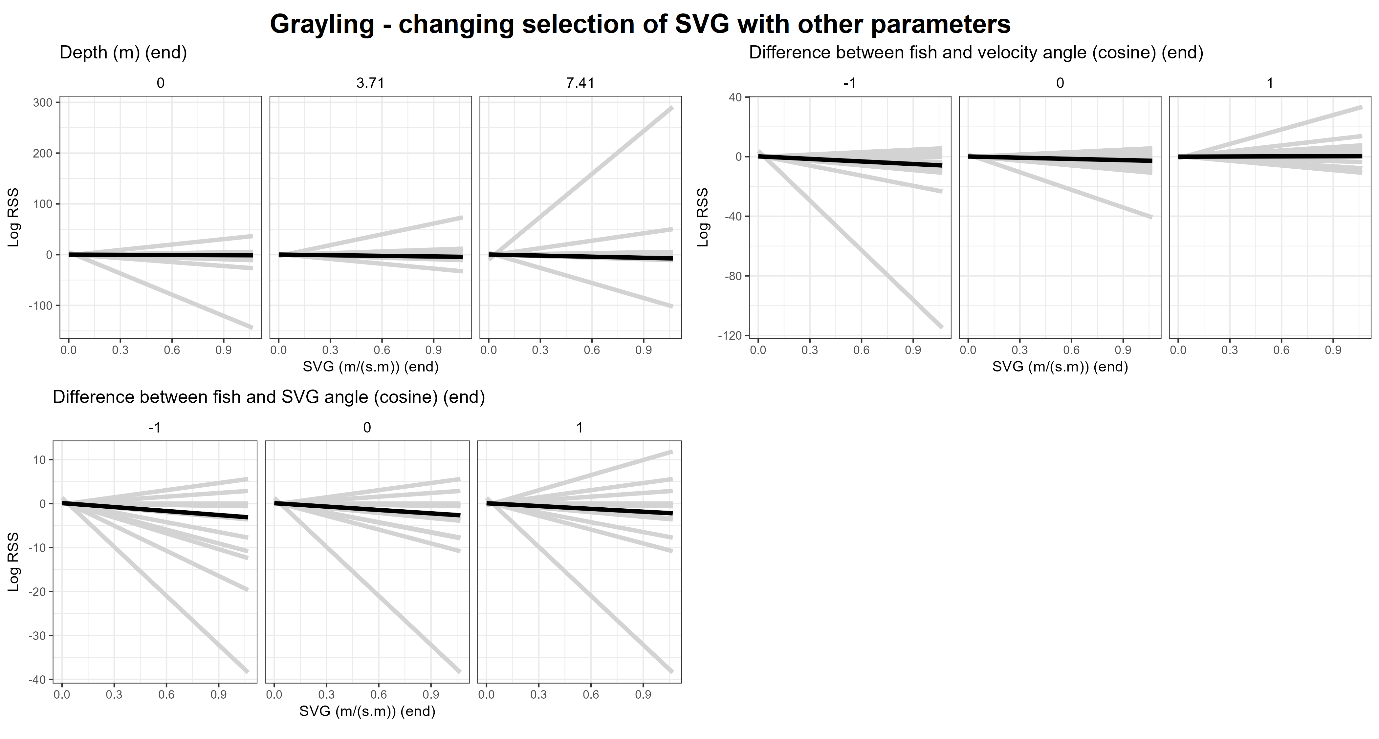
*

*Figure S12. Changing selection of SVG with interacting parameters for grayling. Here, the black line represents the mean value and grey lines show individual relationships. No mean relationships were significant.*

*
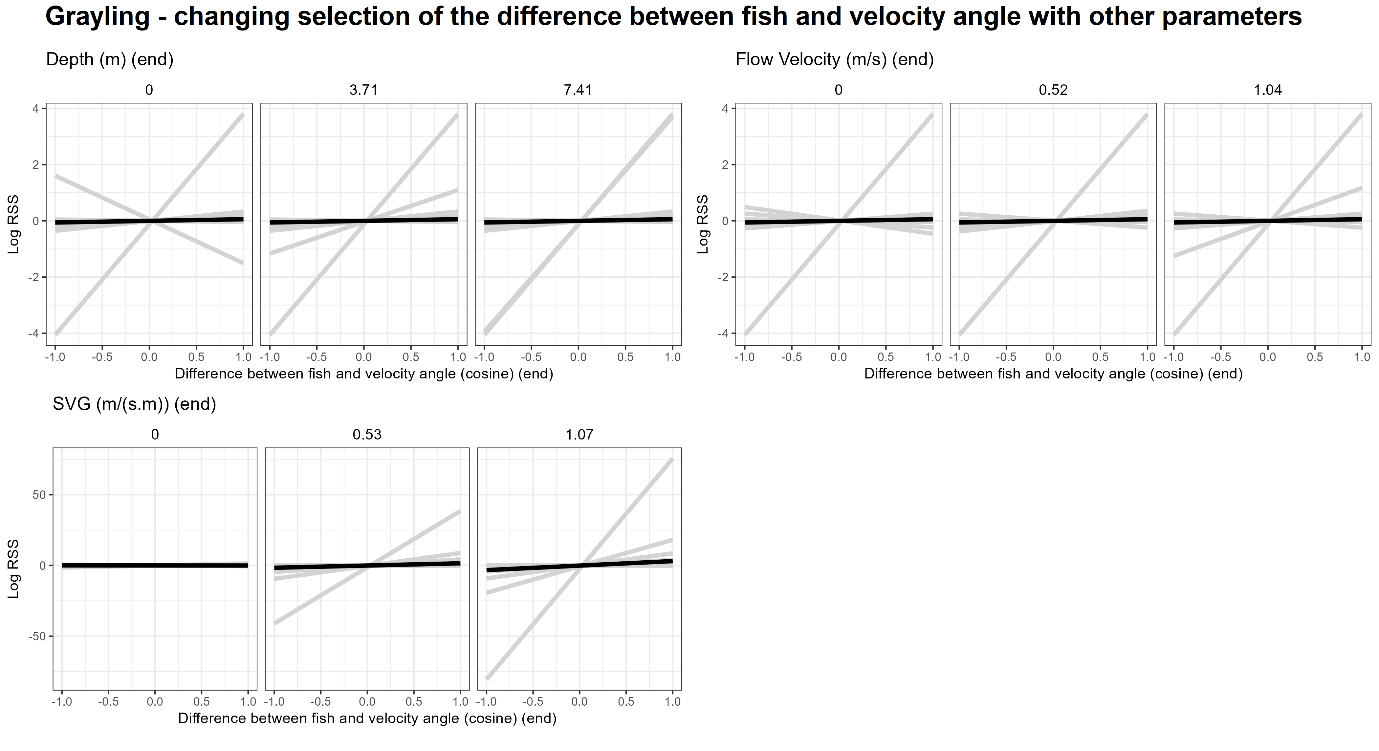
*

*Figure S13. Changing selection of the difference between fish and velocity angle with interacting parameters for grayling. Here, the black line represents the mean value and grey lines show individual relationships. No mean relationships were significant.*

*
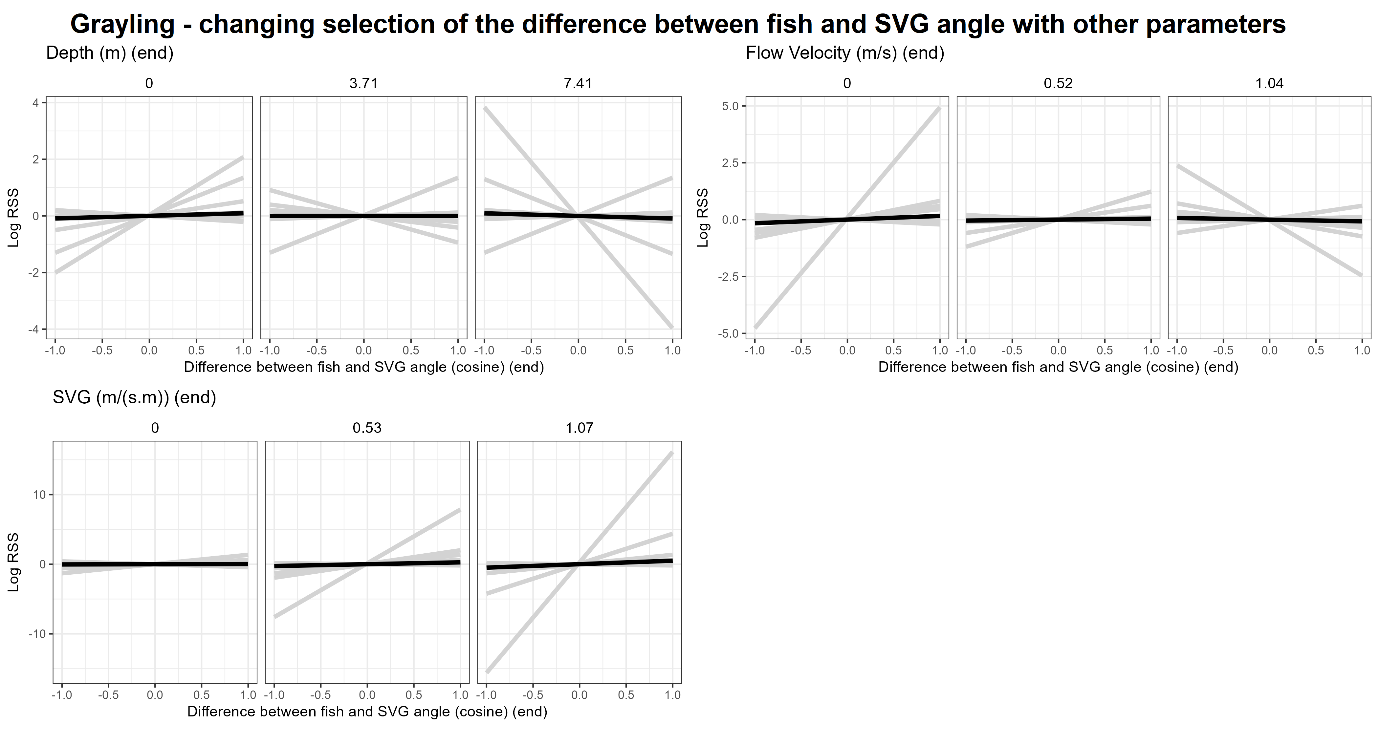
*

*Figure S14. Changing selection of the difference between fish and SVG angle with interacting parameters for grayling. Here, the black line represents the mean value and grey lines show individual relationships. No mean relationships were significant.*


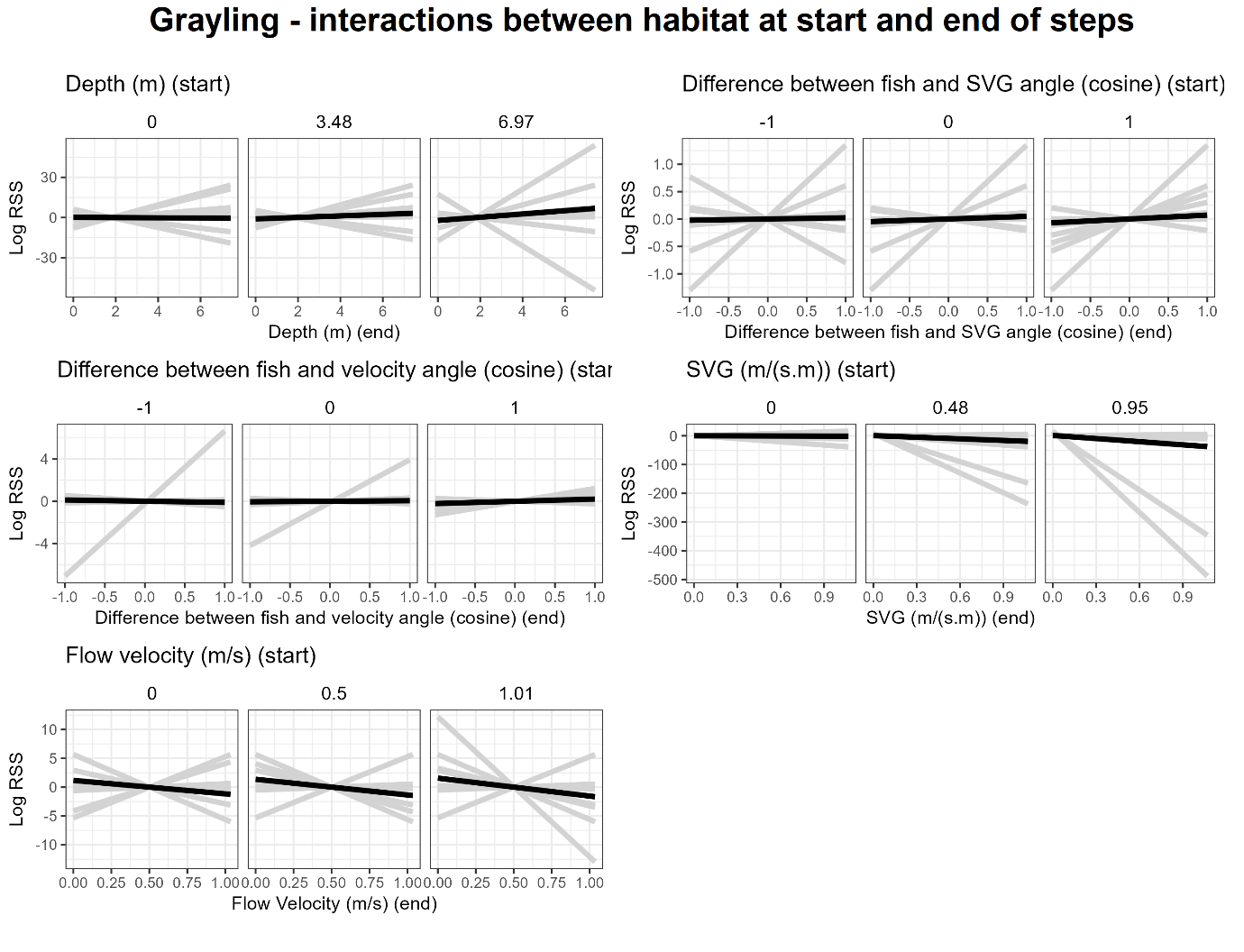


*Figure S15. All interactions between habitat at the start and end of a step for grayling. Here, the black line represents the mean value and grey lines show individual relationships. None of the means were significantly different from zero.*


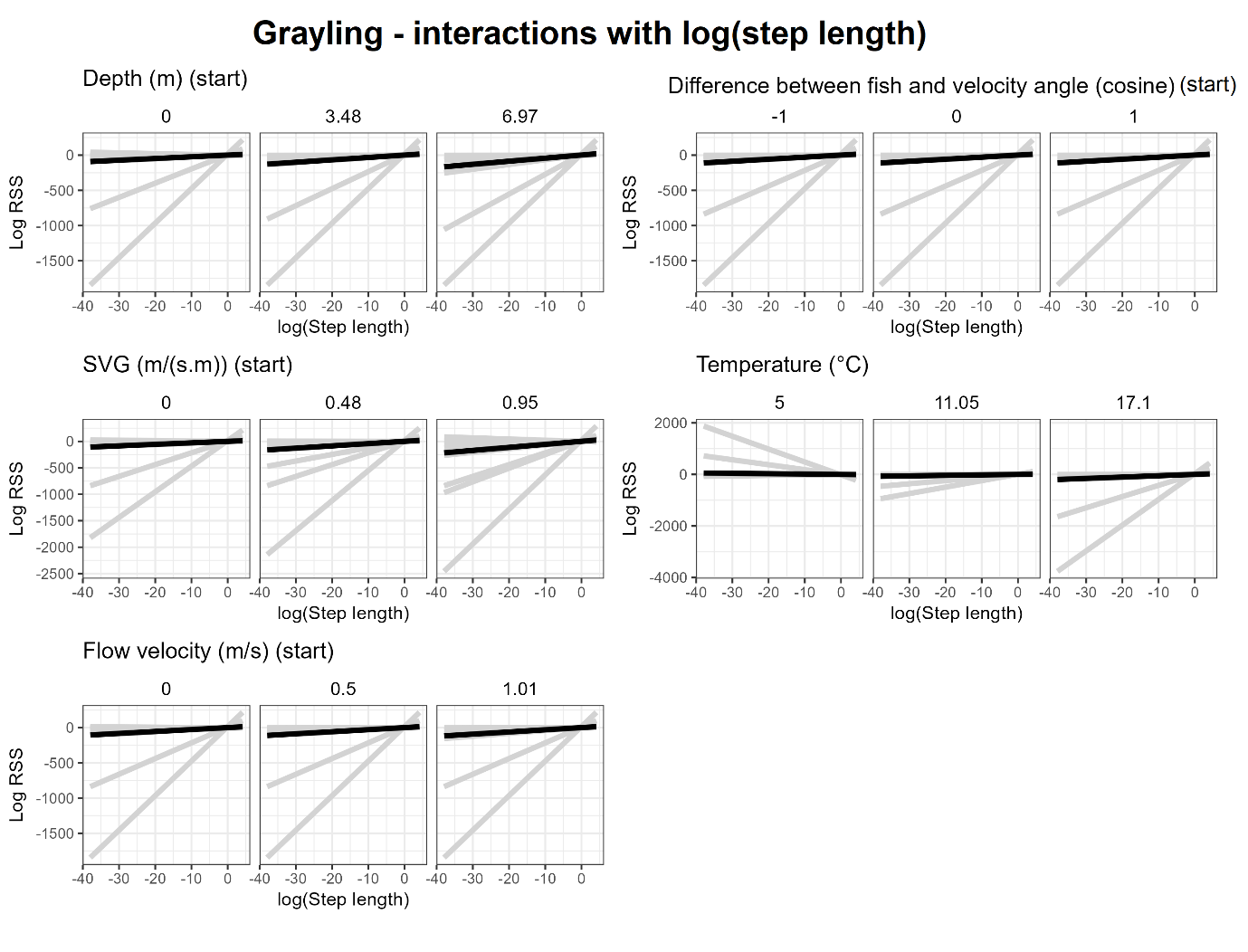


*Figure S16. All interactions between log(step length) and other parameters for grayling. Here, the black line represents the mean value and grey lines show individual relationships. None of the means were significantly different from zero.*

## Coefficients of Population Models

*Table S4. Mean coefficient terms for all terms present in barbel and grayling models. P values are from a one-sample t-test comparing coefficients to a value of zero.*

| Model term | Barbel | | | | | Grayling | | | | | |
| --- | --- | --- | --- | --- | --- | --- | --- | --- | --- | --- | --- |
|  | Mean Coefficient | Standard Deviation | Standard Error | Coefficient of variance | P value | Mean Coefficient | Standard Deviation | Standard Error | Coefficient of variance | P value |  |
| cos(TA) | -0.05206 | 0.1004794 | 0.0230515 | -1.9299175 | 0.036558 | -0.039734 | 0.0860658 | 0.0272164 | -2.1660753 | 0.178321 |  |
| D(end) | 0.399512 | 0.7494321 | 0.1719315 | 1.8758685 | 0.032053 | 0.1768816 | 0.6065382 | 0.1918042 | 3.4290624 | 0.380482 |  |
| D(end):D(start) | 0.050684 | 0.2827969 | 0.0648781 | 5.5795846 | 0.444826 | 0.0874128 | 0.2764235 | 0.0874128 | 3.1622776 | 0.343436 |  |
| D(end):diffSVGang(end) | -0.01573 | 0.0685507 | 0.0157266 | -4.3588989 | 0.330565 | -0.012165 | 0.0384696 | 0.0121651 | -3.1622777 | 0.343436 |  |
| D(end):diffVang(end) | -0.00637 | 0.1818061 | 0.0417092 | -28.556281 | 0.880378 | 0 | 0 | 0 | - | - |  |
| D(end):SVG(end) | 0.138247 | 0.4753235 | 0.1090467 | 3.4382089 | 0.221031 | -0.024233 | 0.205744 | 0.065062 | -8.4903504 | 0.718173 |  |
| D(end):Dusk | 0.010038 | 0.0437537 | 0.0100378 | 4.3588988 | 0.330565 | - | - | - | - | - |  |
| D(end):Night | -0.34424 | 1.5184439 | 0.3483549 | -4.4110613 | 0.336165 | 0 | 0 | 0 | - | - |  |
| diffSVGang(end) | -0.02456 | 0.1072999 | 0.0246163 | -4.3684089 | 0.331591 | 0.0331562 | 0.1537608 | 0.0486234 | 4.6374646 | 0.512468 |  |
| diffSVGang(end):diffSVGang(start) | 0.001103 | 0.0583107 | 0.0133774 | 52.850088 | 0.935178 | 0.0116535 | 0.0368516 | 0.0116535 | 3.1622776 | 0.343436 |  |
| diffSVGang(end):Dusk | 0.006072 | 0.0436047 | 0.0100036 | 7.1811197 | 0.551435 | - | - | - | - | - |  |
| diffSVGang(end):Night | -0.01037 | 0.1150638 | 0.0263974 | -11.093662 | 0.698995 | - | - | - | - | - |  |
| diffVang(end) | 0.251903 | 0.2829814 | 0.0649204 | 1.1233741 | 0.001097 | 0.0413565 | 0.1196623 | 0.0378405 | 2.8934346 | 0.30282 |  |
| diffVang(end):diffVang(start) | 0.039791 | 0.1068224 | 0.0245067 | 2.6846111 | 0.121831 | 0.0824417 | 0.1396814 | 0.0441711 | 1.6943053 | 0.094839 |  |
| diffVang(end):Dusk | -0.02255 | 0.098312 | 0.0225543 | -4.3588989 | 0.330565 | - | - | - | - | - |  |
| diffVang(end):Night | 0.00436 | 0.1204355 | 0.0276298 | 27.62207 | 0.876367 | - | - | - | - | - |  |
| log(SL) | 1.19492 | 2.1255533 | 0.4876354 | 1.7788245 | 0.024721 | 6.4122318 | 14.916356 | 4.7169659 | 2.3262347 | 0.207103 |  |
| log(SL):D(start) | 0.710788 | 0.6703142 | 0.1537806 | 0.9430581 | 0.000212 | 0.4108485 | 0.6077847 | 0.1921984 | 1.4793404 | 0.061257 |  |
| log(SL):diffSVGang(start) | -0.04632 | 0.1311097 | 0.0300786 | -2.8302172 | 0.140924 | - | - | - | - | - |  |
| log(SL):diffVang(start) | -0.04493 | 0.2179249 | 0.0499954 | -4.8500062 | 0.380656 | 0 | 0 | 0 | - | - |  |
| log(SL):SVG(start) | 0.294261 | 0.4990972 | 0.1145007 | 1.6961017 | 0.019278 | 0.2663237 | 0.546316 | 0.1727603 | 2.051323 | 0.157566 |  |
| log(SL):Temp | -1.93182 | 8.6863168 | 1.9927777 | -4.4964498 | 0.345188 | 4.0666506 | 11.922002 | 3.7700681 | 2.9316515 | 0.308795 |  |
| log(SL):Dusk | 0.023941 | 0.0731686 | 0.016786 | 3.0562279 | 0.170915 | - | - | - | - | - |  |
| log(SL):Night | 0.167136 | 0.5417089 | 0.1242765 | 3.2411249 | 0.195368 | -0.084936 | 0.2954449 | 0.0934279 | -3.478433 | 0.38699 |  |
| log(SL):WV(start) | -0.51195 | 0.8202184 | 0.188171 | -1.6021497 | 0.014022 | 0.1468671 | 0.3843794 | 0.1215515 | 2.6171922 | 0.257732 |  |
| SVG(end) | 0.371747 | 0.5473371 | 0.1255677 | 1.4723388 | 0.008373 | -0.111219 | 0.2146245 | 0.0678702 | -1.9297528 | 0.1357 |  |
| SVG(end):diffSVGang(end) | 0.010338 | 0.0450628 | 0.0103381 | 4.358899 | 0.330565 | 0.013225 | 0.041821 | 0.013225 | 3.1622776 | 0.343436 |  |
| SVG(end):diffVang(end) | 0.072646 | 0.118584 | 0.027205 | 1.6323554 | 0.015604 | 0.092329 | 0.1812024 | 0.0573012 | 1.9625735 | 0.141576 |  |
| SVG(end):SVG(start) | -0.00114 | 0.1738529 | 0.0398846 | -152.77086 | 0.977552 | -0.063093 | 0.1995182 | 0.0630932 | -3.1622777 | 0.343436 |  |
| SVG(end):Dusk | 0.026525 | 0.1156186 | 0.0265247 | 4.3588989 | 0.330565 | - | - | - | - | - |  |
| SVG(end):Night | -0.13009 | 0.3504528 | 0.0803994 | -2.693943 | 0.123044 | 0.0803148 | 0.1765397 | 0.0558268 | 2.1980962 | 0.184104 |  |
| WV(end) | 0.575713 | 0.789257 | 0.181068 | 1.3709205 | 0.005191 | -0.57504 | 0.9005683 | 0.2847847 | -1.5660964 | 0.074217 |  |
| WV(end):D(end) | -0.1294 | 0.229841 | 0.0527292 | -1.7761994 | 0.024537 | -0.233517 | 0.2945772 | 0.0931535 | -1.2614817 | 0.033486 |  |
| WV(end):diffSVGang(end) | -0.05372 | 0.0951756 | 0.0218348 | -1.7716157 | 0.024217 | -0.033648 | 0.0751697 | 0.0237708 | -2.2340107 | 0.190577 |  |
| WV(end):diffVang(end) | 0.007488 | 0.1130452 | 0.0259343 | 15.096511 | 0.776083 | 0 | 0 | 0 | - | - |  |
| WV(end):SVG(end) | 0.125082 | 0.1911527 | 0.0438534 | 1.5282161 | 0.01058 | - | - | - | - | - |  |
| WV(end):Night | 0 | 0 | 0 | - | - | 0.0599905 | 0.1897066 | 0.0599905 | 3.1622777 | 0.343436 |  |
| WV(end):WV(start) | -0.13424 | 0.3210692 | 0.0736583 | -2.3916894 | 0.085033 | -0.032744 | 0.1035464 | 0.0327442 | -3.1622776 | 0.343436 |  |

##
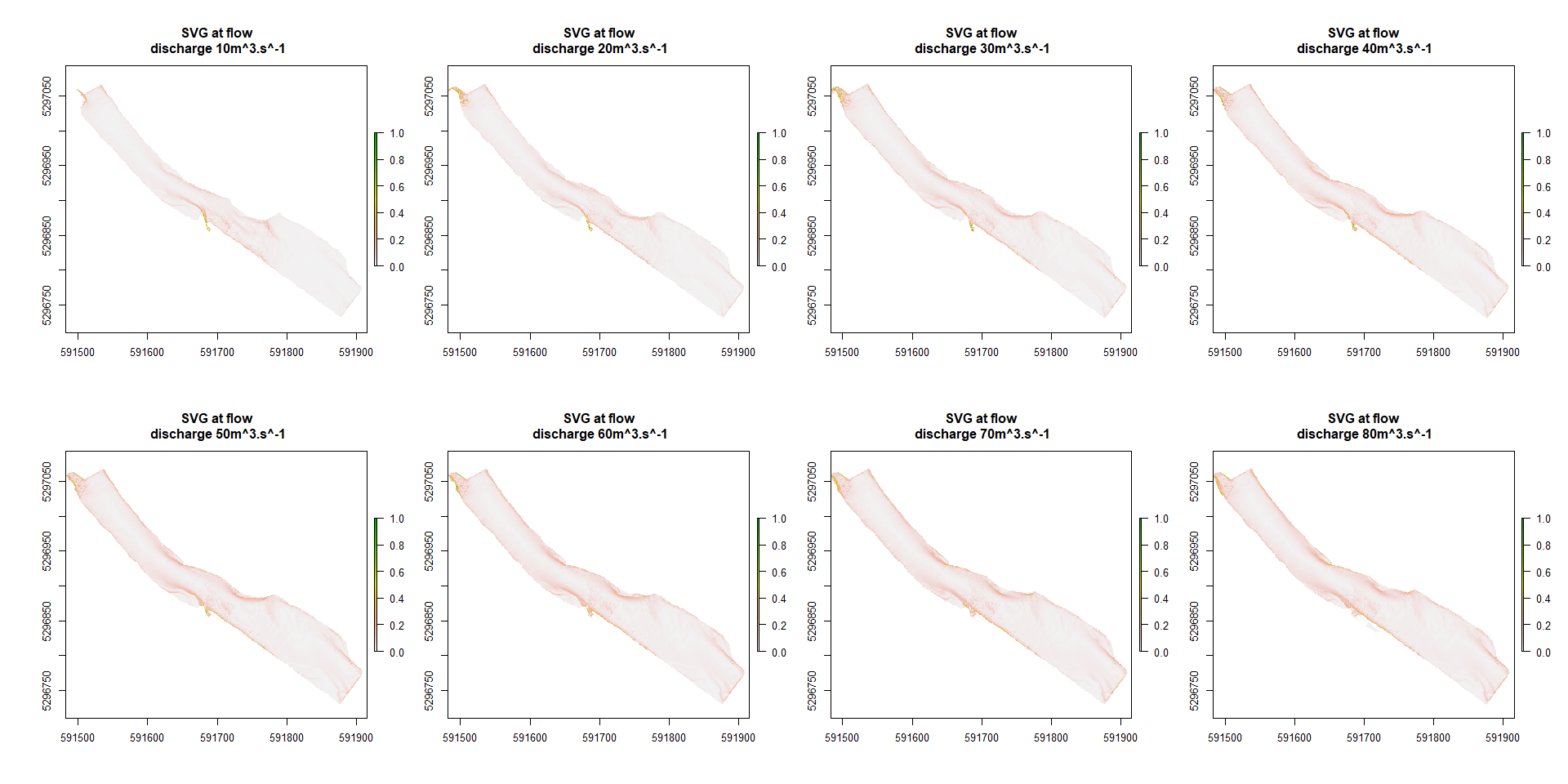
Environmental rasters


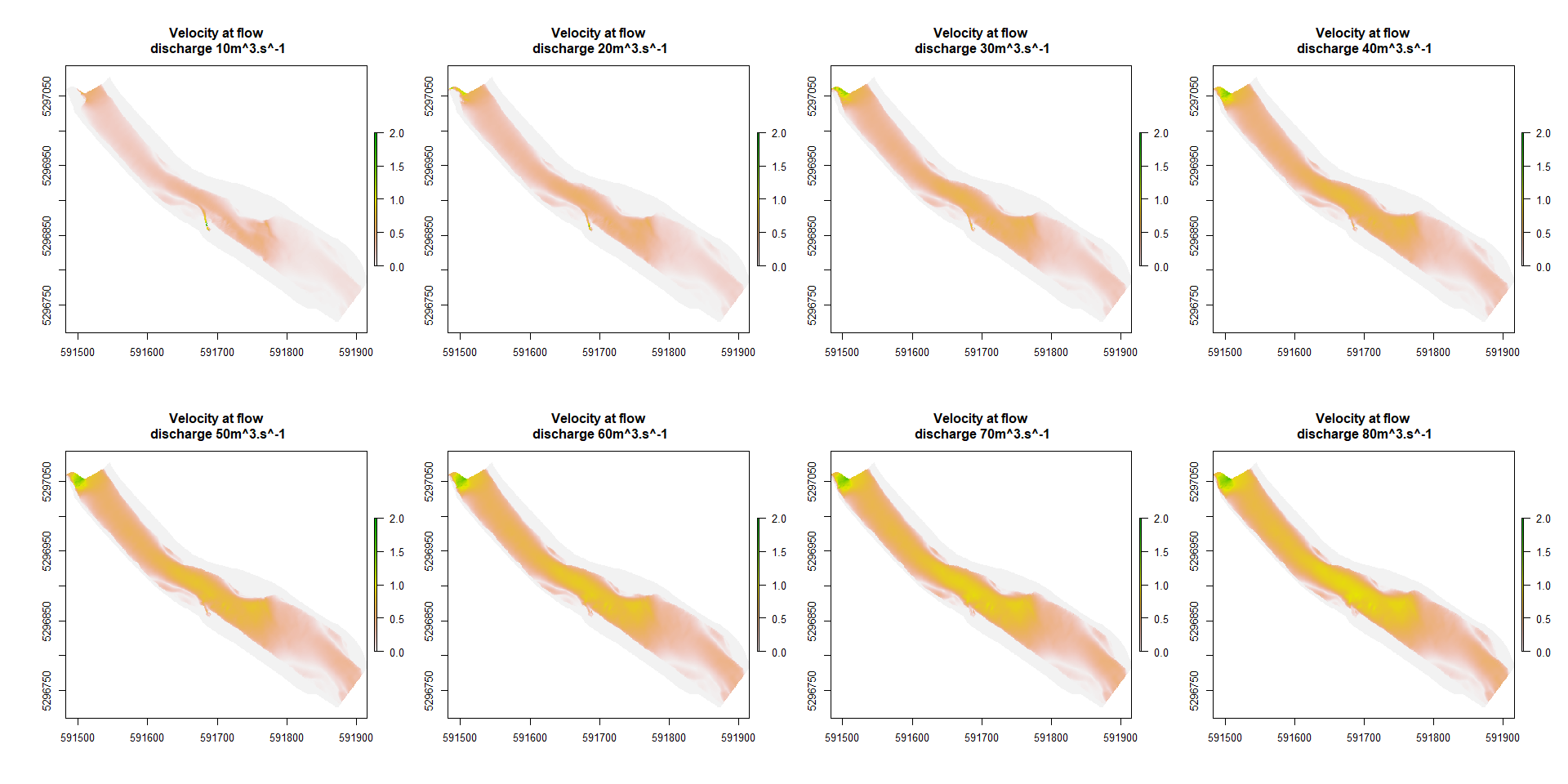

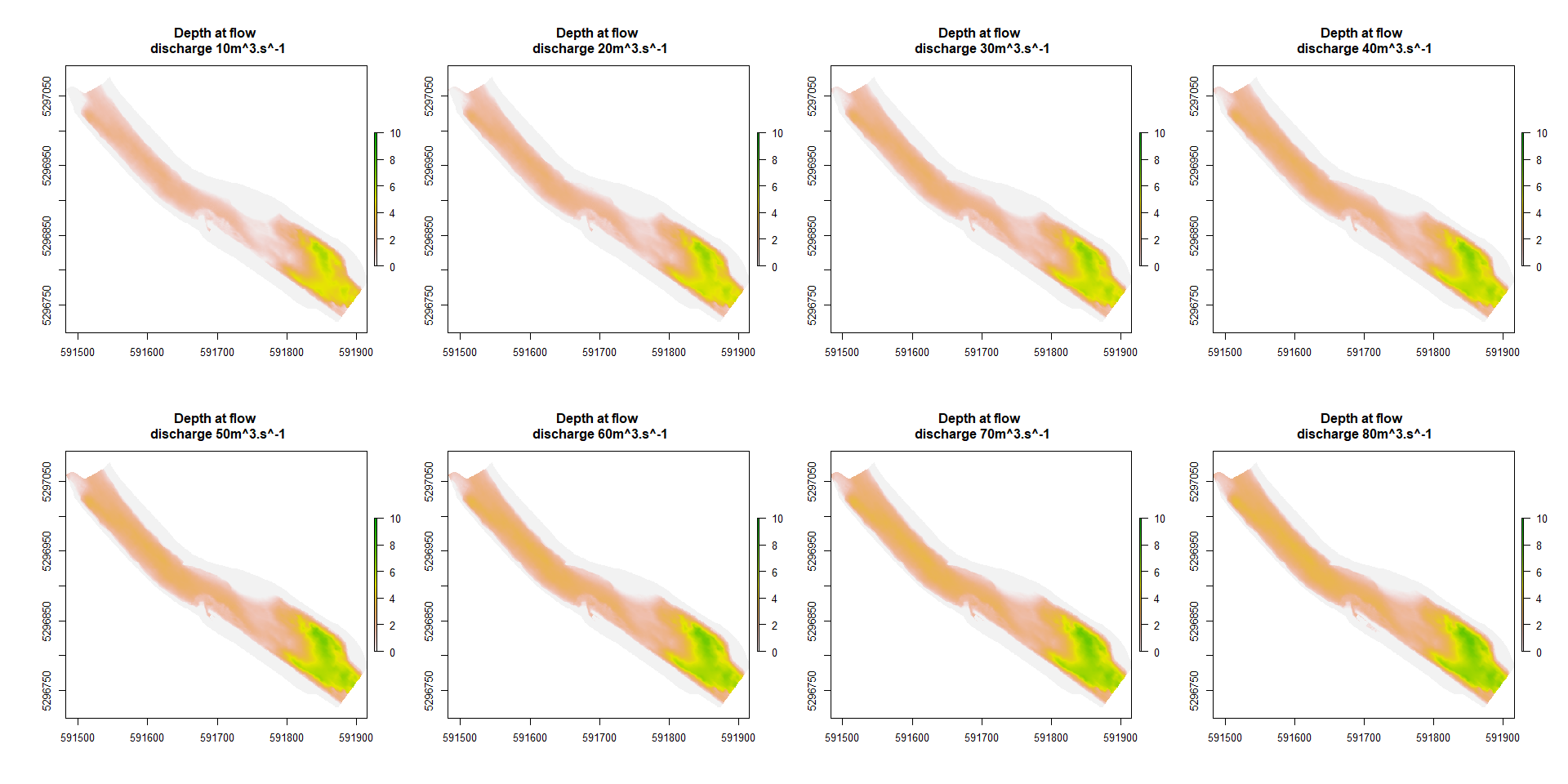


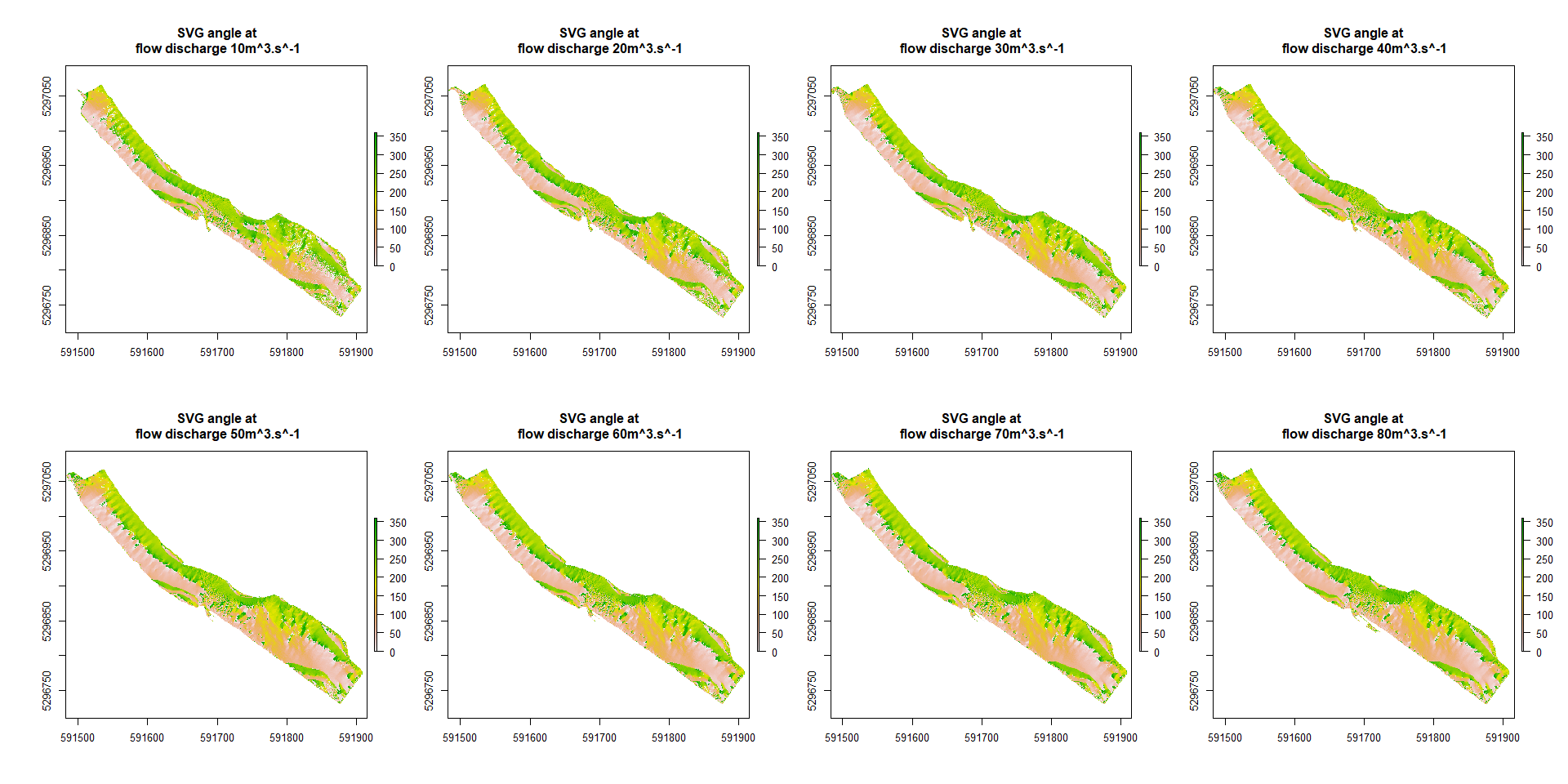

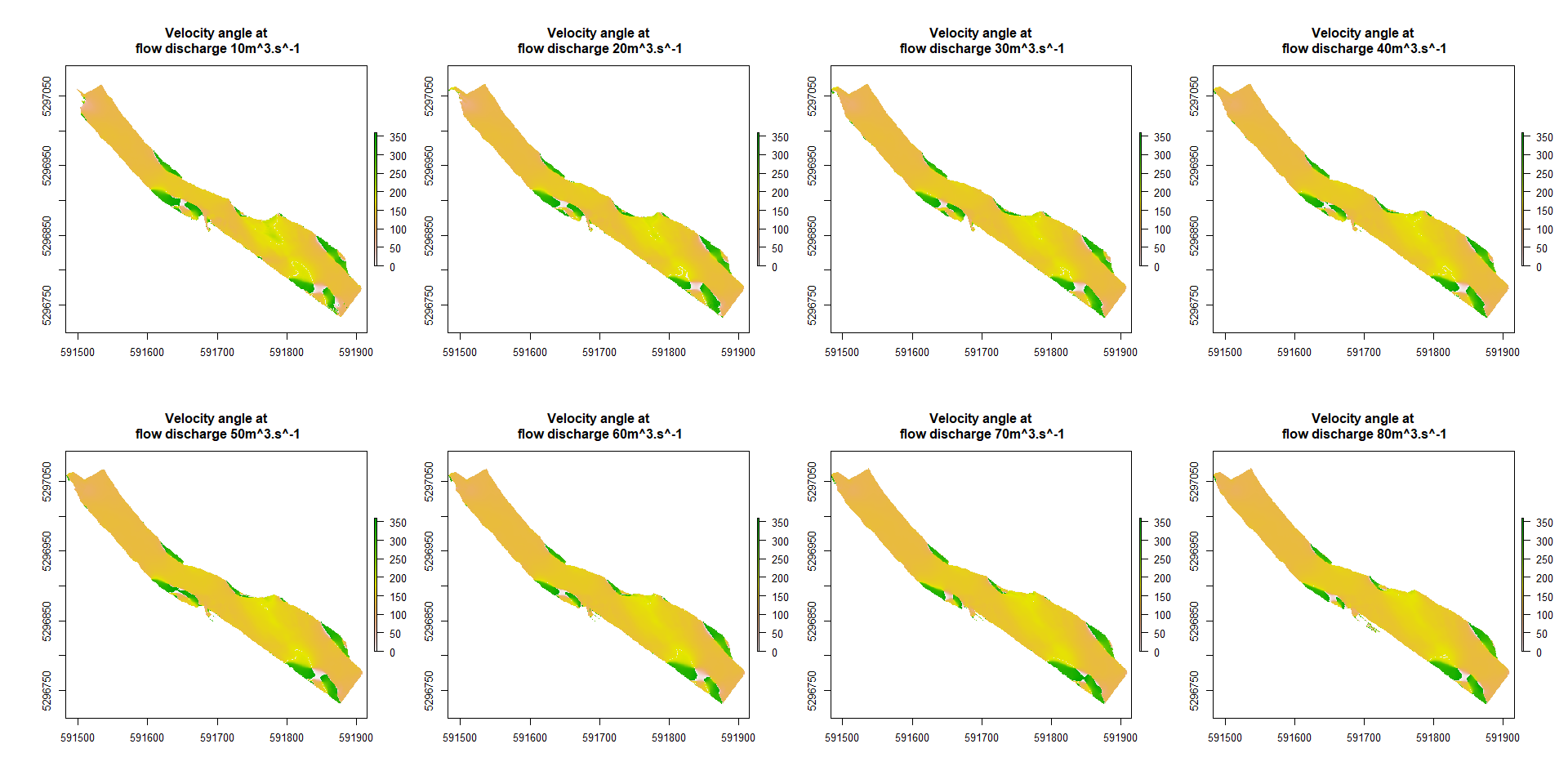

Supplement: Supplementary file 1 — Supplementary Material 1 [file 40462_2023_414_MOESM1_ESM.docx]
